# Conventional and Emerging Drug Targeting Sites in Alzheimer’s Disease and the Role of Translational Informatics in its Diagnosis and Management

**DOI:** 10.2174/011570159X361867250313073624

**Published:** 2025-04-08

**Authors:** Kashif Ali Khan, Muhammad Esa, Zul Kamal, Bashir Ullah, George Perry, Shah Kamal, Shujaat Ahmad, Haya Hussain, Abid Ullah, Muhammad Shafique

**Affiliations:** 1 Department of Pharmacy, Shaheed Benazir Bhutto University, Sheringal 18500, Khyber Pakhtunkhwa, Pakistan;; 2 School of Pharmacy, Shanghai Jiao Tong University, China;; 3 Department of Neuroscience, Developmental and Regenerative Biology, University of Texas at San Antanio one UTSA circle San Antonio, Texas 78249, USA;; 4 Jiangsu King Laboratory for Biodiversity and Biotechnology, College of Life Sciences, Nanjing Normal University, Nanjing 210023, China;; 5 Department of Pharmaceutics, College of Pharmacy, Shaqra University, Shaqra 11961, Saudi Arabia

**Keywords:** Alzheimer’s disease (AD), amyloid-β plaques, novel drug-targeting sites, translational informatics, explainable artificial intelligence (AI), multimodal approaches

## Abstract

Alzheimer’s disease (AD), a neurodegenerative condition, continues to pose significant challenges to modern medicine due to the limited efficacy offered by current therapeutic modalities. With the complex pathophysiology of AD, which includes tau protein accumulation, amyloid-β plaque formation, neuroinflammation, and synaptic dysfunction, novel drug-targeting sites must be identified. This study presents a thorough evaluation of novel drug targeting sites, with a focus on these pathological characteristics as promising therapeutic targets while providing an explanation of their role in the course of the disease. We investigate in detail how neurotoxicity, resulting in synapse failure and cognitive impairment, is caused by tau proteins and amyloid plaques. In addition, the article discusses the increasing evidence that synaptic dysfunction is a major factor in the disease's progression, as well as the significance of neuroinflammation in the pathophysiology of the condition. The review also covers new drug sites such as amyloid-β plaques, tau proteins, and the inhibition of neuroinflammation mediators, in addition to traditional drug sites, including cholinergic and glutamatergic therapeutic targets. Lastly, we discuss the role of translational informatics involving data modeling, predictive analytics, explainable artificial intelligence (AI), and multimodal approaches for the management and prediction of AD. This article will serve as a guide for future research efforts in the fields of neuroscience, neuropharmacology, drug delivery sciences, and translational informatics.

## 1 INTRODUCTION

Alzheimer’s disease (AD) is a neurological condition characterized by memory loss, cognitive impairments, and behavioral changes. It is the most common form of dementia, a term used to describe a group of symptoms that impair a person’s ability to function properly in daily life [1]. The disease affects more than 6.5 million people in the United States aged 65 and older. Out of approximately 55 million people with dementia globally, 60% to 70% are estimated to have AD. The cost of caring for individuals with AD is substantial, including both medical expenses and informal care provided by family members and caregivers [2]. Early research shows that the morbidity of AD increases up to 10-50% over the age of 65 [3]. According to some estimates, roughly 65.7 million individuals will suffer from dementia by 2030, and this figure could more than quadruple to 115.4 million by 2050 if effective treatments are not developed [4].

**Fig. (1):**
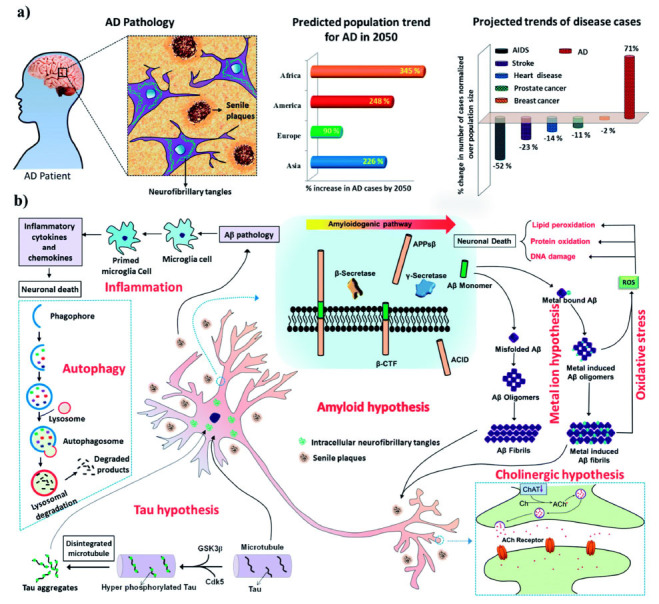
(**a**) Shows characteristic features of the pathology of AD in the human brain and recent statistics showing the rapid rise of AD as a global epidemic. (**b**) Multiple pathological pathways of AD. Copyrights^®^ Royal Society of Chemistry 2018 [22].

Some of the main features of AD include the formation of protein clumps called amyloid-β plaques between the nerve cells in the brain, which are believed to interfere with communication and damage the brain cells, as well as neurofibrillary tangles, which are twisted fibers of tau proteins that accumulate inside the nerve cells in the brain. These accumulations interrupt the transport of nutrients and other substances between the cells, leading to damage in brain tissue. Additionally, the disease is characterized by brain atrophy, referring to the shrinkage of brain tissues due to the loss of nerve cells and their connections (Fig. **1**). This atrophy affects areas of the brain responsible for memory, language, reasoning, and social skills [5].

The symptoms of AD are highly individualized and depend on the stage of the disease. The most noticeable symptom is memory loss, which impairs the ability to remember recent events, conversations, names, and places while also hindering the capacity to learn new information and recall past memories [6]. Thinking and reasoning capabilities decline, affecting concentration, problems-solving, decision-making, planning, and the ability to understand numbers, words, and concepts. Communication and language are also disturbed, leading to difficulty finding the right words, expressing thoughts, following conversations, and impairing the ability to read, write, and speak clearly. Vision and spatial awareness deteriorate, impacting the capability to perceive depth, distance, shapes and recognize faces, objects, and colors [7]. Behavior and personality undergo significant changes, affecting the mood, emotions, and social skills of the patient. These changes can cause anxiety, depression, agitation, aggression, paranoia, hallucinations, and delusions. Apathy, withdrawal, and a loss of interest in activities are also common [8].

The causes of AD are not yet fully understood, but some factors have been identified that increase the risk of the disease. The risk of developing AD increases with age, especially after the age of 65. However, it is not a normal aspect of aging and can affect younger individuals as well [9]. Genetic factors may also influence the risk of developing the disease. The most well-known gene is apolipoprotein E (*APOE*), which comes in three forms: *APOE2, APOE3*, and *APOE4*. Possessing one or two copies of *APOE4* increases the risk, while having *APOE2* may lower it. However, not everyone with *APOE4* will develop the disease, and not everyone without *APOE4* is protected from it [10]. Family history is also a significant risk factor; having a parent, sibling, or child with the disease may increase the risk of developing it oneself. This increased risk may be due to genetics, environmental factors, or a combination of both. Some lifestyle and environmental factors may lower the risk of developing the disease, including physical activity, mental stimulation, social engagement, a healthy diet, and avoiding alcohol, smoking, and head injuries. Diabetes, high blood pressure, high cholesterol, and obesity may increase the risk of developing the disease [11].

Although there is no specific test to diagnose AD, doctors employ various methods to find out possible causes of dementia and assess the degree of cognitive impairment. A comprehensive medical history is one such method and involves questioning the patient about the symptoms, duration, progression, and impact of the condition, as well as their lifestyle and medical history. Doctors also perform physical and neurological examinations, assessing the patient’s vital signs, reflexes, coordination, balance, and sensory functions. It also evaluates other diseases that may affect the brain, such as stroke, infection, or tumor [12]. Cognitive and neuropsychological tests are also conducted to evaluate the patient’s memory, attention, language, reasoning, and other mental abilities, which helps determine the type and severity of dementia and identify the affected areas of the brain [13]. Laboratory tests like blood, urine, and cerebrospinal fluid analysis can assist in identifying infections, vitamin deficiencies, hormonal imbalances, or genetic markers linked with AD [14]. Brain imaging techniques like CT scan, MRI, positron emission tomography, and single photon emission computed tomography (SPECT) allow visualization of the structure, function, and blood flow of the brain. These imaging methods provide critical insights into brain health and help detect abnormalities associated with AD [15].

AD has a profound impact on individuals, their families, and society as a whole. It leads to a gradual decline in cognitive functioning, affecting memory, thinking, and social skills. The disease disrupts daily life, causing trouble with everyday tasks. People with AD may repeat statements and questions over and over, forget conversations, appointments, or events, and misplace items frequently [16]. The disease can lead to behavioral changes, including changes in personality, such as becoming aggressive, demanding, or suspicious of others. As the disease progresses, some people may become anxious, angry, or violent. In advanced stages, it causes severe loss of brain functions, which can lead to dehydration, malnutrition, or infections, ultimately resulting in death [17].

AD is a degenerative brain disorder that affects memory, thinking, and behavior. It is the leading cause of dementia, impacting millions of individuals globally. There is currently no treatment that cures or significantly slows the progression of AD, but several medications and therapies can help manage symptoms and improve the quality of life for people living with the condition and their caregivers [17]. Certain medications may enhance memory, thinking, and behavior in patients with the disease. These medications work by modifying the levels of specific chemicals in the brain, such as acetylcholine and glutamate. Commonly used medications include cholinesterase inhibitors (*e.g*., donepezil, rivastigmine, and galantamine) and NMDA receptor antagonists (*e.g*., memantine) [18]. Non-drug therapies are interventions that help to stimulate the cognitive, emotional, and social aspects of patients with the disease, reducing stress, anxiety, depression, and agitation. Some examples of such therapies are meditation, cognitive stimulation, reminiscence therapy, music therapy, art therapy, and animal-assisted therapy. It is important to provide supportive care to the patient and caregivers, including necessary information, education, guidance, and resources to cope with the disease [19]. Such care includes support groups, counseling, respite care, home care, and hospital care. These therapies provide some assistance to the patient but are not highly effective [20]. The necessity for effective treatment of AD is as high as ever, as there is an expected rise in the number of people affected by the condition in the coming years. Researchers are working hard to find new ways to prevent, diagnose, and treat the disease, as well as to improve the care and support for people living with it and their families [21].

In the literature, a lot of research articles can be found on AD, conventional drugs, emerging therapeutic drug targets, and the role of translational informatics such as data modeling, predictive analytics, explainable artificial intelligence (AI), and multimodal approaches for the treatment, management, and prediction of AD. Each study requires attention in order to get a meaningful outcome. However, the results are scattered, leaving room for a review article to explain the role of emerging therapeutic drug targets and discuss the role of translational informatics in the prediction, diagnosis, onset, and management of AD. In this paper, we reviewed the conventional drug-targeting approaches for treating AD. Additionally, we present the mechanistic overview of various studies showcasing the emerging drug targeting sites and novel drugs in clinical trials or already approved by the US Food and Drug Authority (FDA) in the past decade. Lastly, we focused on the role of translational informatics in AD management and analysis. Each step is supported by a preclinical study from the literature, and we hope this review will help researchers gain interesting insights regarding drug targeting sites and translational informatics.

## 2 CURRENT TREATMENT LANDSCAPE

Currently, there are two main types of drug sites employed in the treatment of AD: cholinergic and glutamatergic. These drug targets correspond to the neurotransmitters acetylcholine and glutamate, which play key roles in memory and learning processes in the brain.

### 2.1 Cholinergic Drug Sites

Cholinergic drug sites are receptors that respond to acetylcholine and are classified into two main types: muscarinic and nicotinic. Muscarinic receptors are G-protein-coupled cholinergic receptors that regulate various cellular processes, such as enzyme activity, ion channel opening, and gene expression. In contrast, nicotinic receptors are ionotropic receptors that form channels allowing the influx of sodium and calcium ions, leading to depolarization and neurotransmitter release. These receptors are targeted by cholinesterase inhibitors, which act by preventing the breakdown of acetylcholine, a chemical messenger depleted in the brain due to AD. By increasing acetylcholine levels, these drugs may improve memory and cognition in individuals with mild to moderate AD [23].

**Fig. (2):**
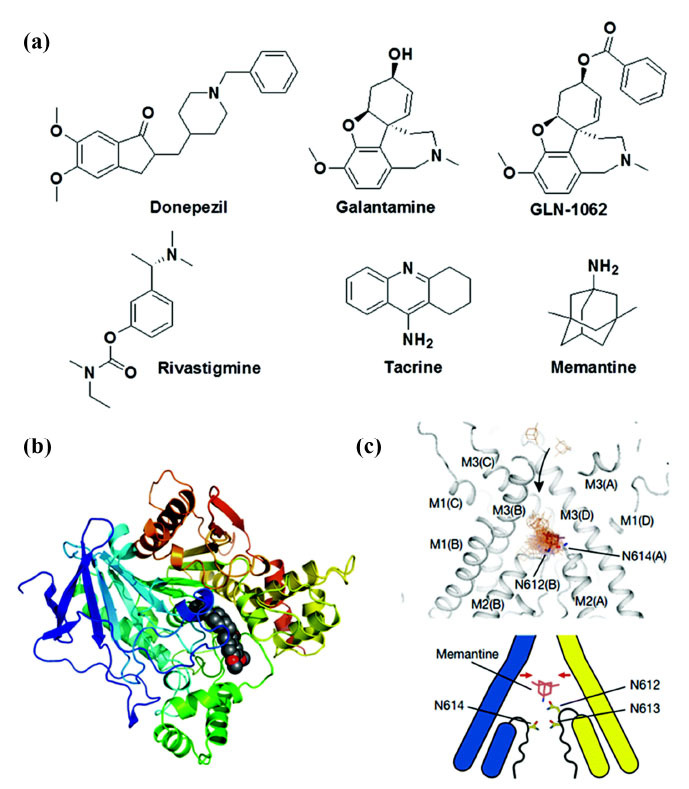
(**a**) Cholinergic inhibitors and NMDA antagonists for treating AD. Molecular docking of donepezil (**b**) and memantine (**c**) with AChE and NMDA receptors, respectively. This figure has been adapted from [27]. Copyrights^®^ Nature 2018.

In AD, cholinergic neurons are lost, and levels of acetylcholine in the brain are reduced, especially in the cerebral cortex and hippocampus, regions critical for cognition. This loss impairs synaptic transmission and plasticity and results in cognitive decline and dementia [24]. Targeting cholinergic receptors involves using drugs that either increase acetylcholine availability or directly activate the receptors. The most common drugs that increase acetylcholine levels are cholinesterase inhibitors, such as galantamine, rivastigmine, and donepezil (Fig. **2**). These drugs work by inhibiting the breakdown of acetylcholine, thereby prolonging its action in synapses. On the other hand, drugs that directly activate the receptors include nicotinic agonists, such as nicotine, varenicline, and galantamine. These drugs mimic acetylcholine’s effects and stimulate the receptors [25].

Targeting cholinergic drug receptors in AD can be somewhat effective, but there are some notable limitations. These drugs do not address the underlying cause of the disease. Additionally, their effects on cognitive symptoms are modest and variable. They do not prevent the progression of the disease or the decline of other functions, such as language, visuospatial skills, and behavior. Furthermore, there are some side effects, including nausea, vomiting, diarrhea, abdominal pain, weight loss, and bradycardia, which can limit their tolerability and adherence. They may also interact with other drugs possessing anticholinergic properties, such as some anti-depressants, antihistamines, antipsychotics, and antispasmodics, which can worsen cognitive impairment or cause additional adverse effects [26].

### 2.2 Glutamatergic Drug Sites

Glutamatergic drug sites in AD refer to targets modulated by drugs that influence the activity of glutamate, the primary excitatory neurotransmitter in the brain. Glutamate is involved in cognitive functions such as memory and learning. However, abnormal levels (too low or too high) may lead to neurotoxicity [28]. Glutamate receptors are located at both synaptic sites (where they mediate synaptic transmission and plasticity) and extrasynaptic sites (where they regulate neuronal excitability and survival). Glutamatergic drug sites are targeted by N-methyl-D-aspartate (NMDA) receptor antagonists, such as memantine. These drugs function by blocking excessive glutamate activity, which can lead to brain damage in AD. By reducing glutamate overstimulation, these drugs help protect brain tissue and delay the progression of cognitive decline in patients with moderate to severe AD [29].

An NMDA receptor site is a specific location on the surface of a neuron where NMDA receptors, a subtype of glutamate receptors, are expressed. NMDA receptors, along with glutamate, are essential for memory and learning processes. In AD, an imbalance in glutamate signaling results in synaptic dysfunction and neural death, which are the two hallmark features of the disease. Targeting these receptor sites aims to modulate receptor activity and restore glutamate signaling balance [28, 30]. Currently, memantine is the only approved drug targeting NMDA receptor sites for AD. It is prescribed for moderate to severe cases. Memantine selectively blocks the function of extrasynaptic NMDA receptors, protecting neurons from glutamate toxicity and excitotoxicity [31]. Targeting NMDA receptor sites in AD has several limitations. NMDA receptor antagonists exhibit modest and inconsistent effects on cognitive symptoms and do not halt disease progression or prevent declines in other functions. Additionally, they may cause side effects like dizziness, confusion, headache, constipation, and agitation, which can affect tolerability and adherence. These drugs also fail to address the underlying causes of AD and may interact with other NMDA receptor-active medications, potentially exacerbating cognitive impairment or causing additional adverse effects [32].

## 3 EMERGING DRUG SITES

With current treatments proving ineffective in halting neural degeneration or slowing AD progression, novel drug targets are being explored to develop therapies that modify the disease course. One of the most promising approaches focuses on the pathological hallmarks of AD, including amyloid-β plaques, tau protein aggregation, neuroinflammation, and synaptic dysfunction. These targets offer the potential for more effective treatments that could slow or reverse disease progression by addressing the underlying mechanisms of neurodegeneration.

### 3.1 Amyloid-β Plaques

Amyloid-β plaques, abnormal protein deposits in the brain, are key targets for AD therapy. These plaques, along with neurofibrillary tangles, are two of the hallmarks of the disease [33]. Amyloid-β plaques are thought to interfere with the communication and function of neurons, leading to memory loss and cognitive abilities. However, the exact role of the disease process is still unclear, as some people may have high levels of amyloid-β in their brains without showing any symptoms of dementia [34]. There are different types of amyloid-β plaques, depending on the molecular structure and length of the amyloid-β protein. The most common and toxic form is amyloid-β 42, which has 42 amino acids and forms S-shaped filaments that aggregate into plaques. Different types of amyloid plaques may be associated with different types of AD, such as sporadic or familial forms [35].

Targeting these plaques represents a novel approach to the treatment of AD. These plaques are extracellular deposits of aggregated peptides formed by the breakdown of amyloid-β protein precursor (AβPP) by β- and γ-secretases. These plaques are thought to trigger a cascade of pathological events, such as neuroinflammation, oxidative stress, synaptic dysfunction, and tau hyperphosphorylation, leading to neuronal loss and cognitive impairment in the disease [36]. To address this issue, various anti-amyloid-β therapies have been developed or are under development, with the aim of reducing the production, aggregation, or accumulation of amyloid-β plaques in the brain. Some of these therapies include:

1. Monoclonal antibodies bind to amyloid-β peptides and promote their clearance or prevent their aggregation. Examples of such antibodies are aducanumab, lecanemab, and donanemab, which have shown positive results in clinical trials [37].
2. BACE inhibitors block the activity of β-secretase, the enzyme that initiates the cleavage of AβPP to generate amyloid-β peptides. Examples of BACE inhibitors are verubecestat and atabecestat, which have failed to show efficacy in clinical trials [38].
3. Gamma-secretase modulators modulate the activity of γ-secretase, the enzyme that completes the cleavage of AβPP to generate amyloid-β peptides. Examples of gamma-secretase modulators are semagacestat and avagacestat, which have also failed to show efficacy in clinical trials [39, 40].
4. Vaccines that induce an immune response against amyloid-β peptides and facilitate their clearance or prevent their aggregation. Examples of such vaccines are AN1792 and CAD106, which have shown some benefits in clinical trials [41, 42].

### 3.2 Tau Proteins

**Fig. (3):**
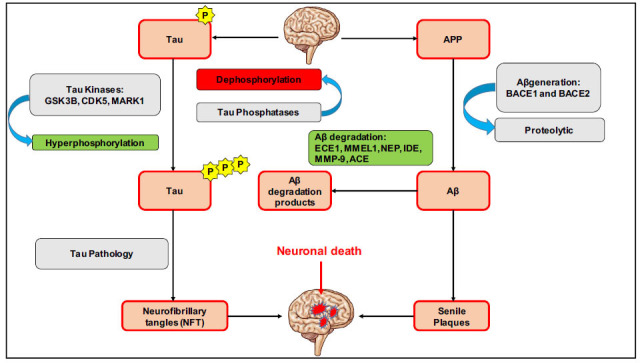
Mechanistic overview of neuronal death through the generation of tau and amyloid-β plaques with subsequent enzymatic actions and/or phosphorylation mechanisms.

Tau proteins are a type of protein that normally helps stabilize the structure and function of neurons, which are brain cells that transmit information. These are the main components of the neurofibrillary tangles that form inside the neurons of AD patients. In normal neurons, it stabilizes microtubules that support the structure and function of neurons [43]. However, in AD, tau is abnormally phosphorylated and aggregates to form filaments called neurofibrillary tangles within neurons. These tangles impair communication and neuron survival, leading to memory loss and cognitive decline (Fig. **3**) [44]. Targeting tau protein aggregation is a potential therapeutic strategy for AD treatment, as it is a major pathological feature of the disease. There are different approaches to target tau aggregation in AD, including:

1. Inhibiting the enzymes that phosphorylate tau, which can increase its propensity to aggregate. For instance, a previous study reported the synthesis of 2,4-thiazolidinedione derivatives with the potential to reduce *AcPHF6* aggregation by 60% and 80% at 10 μM, respectively, and inhibit the phosphorylating tau kinase GSK-3β and the tau aggregation process. Additionally, even at 50 μM, the test compounds demonstrated minimal hepatotoxicity against the human hepatoma cell line (HepG2) and a satisfactory cellular safety profile with excellent blood-brain barrier permeability (>3.07 × 10^-6^ cm/s) tested though parallel artificial membrane permeability test (PAMPA). Specifically, both K18 and full-length tau aggregations were inhibited by compound (Z)-5-((5-Methoxy-1-methyl-1H-indol-3-yl) methylene) thiazolidine-2,4-dione, which also improved cell survival in a neurodegenerative cell model produced by okadaic acid [45].
2. Activating the enzymes that dephosphorylate tau which helps in reducing tau aggregation. For instance, a recent work described the development of a tau dephosphorylating-targeting chimera (DEPTAC) and produced a novel chimera D14, which demonstrated minimal cytotoxicity and excellent efficacy in lowering tau phosphorylation in both cell and tauopathy mice models. Additionally, D14 enhanced the cognitive abilities of tauopathy mice and reduced neurodegeneration in primary cultured hippocampus neurons treated with harmful tau-K18 fragments [46].
3. Blocking the interaction between tau molecules leads to aggregation, which prevents tau aggregation. A recent study documented the development of a small-molecule inhibitor of the Tau-SH3 interaction. SRI-42667, the optimized compound, attached to Tau and prevented endogenous Tau-SH3 interaction in neurons while leaving Tau-tubulin interactions unaffected. The prevention of Aβ-induced increases in neuronal firing by SRI-42667 and a peptide inhibitor of Tau-SH3 contacts indicates Tau-SH3 interactions performed an essential role in AD-related network hyperexcitability [47].
4. Enhancing the clearance of tau aggregates by the immune system or proteasome. One such example is neurotransmitter-derived lipidoid (NPD), which has recently been reported to promote the clearance of tau aggregates and help in cognitive recovery for AD. Intravenous injection of NPD led to a significant improvement in the cognitive function of the AD mice without any remarkable abnormalities, thereby supporting its clinical development [48].
5. Immunization with antibodies that recognize and neutralize tau aggregates [49]. Based on the mentioned approach, the latest study reported the development of a pentavalent nanoparticle vaccine comprising two Aβ peptides (1-14 and pyroglutamate pE3-14) and three tau peptides (centered on phosphorylated pT181, pT217, and pS396/404). When mice were given separate epitopes *via* intramuscular injection, the production of slightly cross-reactive antibodies occurred; however, when five antigens were shown simultaneously (referred to as the “5-plex”), antibodies were produced against every epitope without immunological interference. Neurofibrillary tangles and plaques were identified by post-immune sera from patient AD brain tissue. 3xTg-AD mice that received the vaccine on a preventive dosing regimen showed enhanced cognitive performance and inhibition of tau and amyloid proteins. Immunization was well tolerated, and neither the central nervous system nor the peripheral nervous system had chronic inflammatory reactions or antigen-specific cellular reactions [50].

### 3.3 Neuroinflammation

Neuroinflammation refers to the activation of immune cells, such as microglia and astrocytes, in response to various stimuli like infections, injuries, or abnormal protein accumulation. This phenomenon is one of the key features of AD [51]. Some studies suggest that neuroinflammation may have both harmful and beneficial effects on disease progression, depending on the type, timing, and location of the inflammatory response [51]. Some of the molecular pathways and mediators involved in neuroinflammation during AD are presented in Fig. (**4**) and discussed as follows:

1. The transcription factor nuclear factor kappa B (NF-κB) controls the expression of several inflammatory genes, such as adhesion molecules, chemokines, and cytokines.
2. p38 mitogen-activated protein kinases (p38 MAPK), a kinase that activates NF-κB and other inflammatory factors, such as COX-2 and iNOS3.
3. Akt/MTOR, a pathway that regulates cell survival, growth, and metabolism, and is deregulated in AD.
4. Caspase, a family of proteases that mediate apoptosis and inflammation and are activated by amyloid-β plaques and neurofibrillary tangles.
5. Nitric oxide (NO), a free radical that modulates synaptic transmission, blood flow, and inflammation.
6. COX, an enzyme that catalyzes the synthesis of prostaglandins, which are involved in pain, fever, and inflammation and are increased in AD.
7. Tumor necrosis factors (TNF), interleukins, and chemokines, which are cytokines regulating the communication and activation of immune cells [52].

Some potential strategies that may be used to target neuroinflammation in AD include (a) Peroxisome proliferator-activated receptor gamma (PPAR-γ) antagonists, which are drugs that activate a nuclear receptor that modulates inflammation and metabolism and may enhance the clearance of amyloid-β plaques and reduce the production of interleukins-1β. (b) Gut microbiota modulation, which involves the manipulation of the composition and functions of intestinal bacteria, can influence brain inflammation and immunity through the gut-brain axis. (c) Anti-inflammatory drugs, such as NSAIDs, steroids, and biologics, can inhibit the production or action of inflammatory mediators, such as COX, TNF, and ILs. And, (d) Immunotherapy, which uses antibodies or vaccines to target and remove Aβ plaques or neurofibrillary tangles from the brain and modulates the immune response [53].

### 3.4 Synaptic Dysfunction

Synaptic dysfunction refers to the impairment of communication between neurons, which is essential for learning and memory. Synapses are the connections between neurons that allow them to communicate and form neural networks. Synaptic dysfunction is one of the earliest and most prominent features of AD [54]. AD is thought to be caused by several factors, such as the accumulation of toxic forms of amyloid-β and tau proteins, activation of microglia and inflammatory responses, disruption of neuronal metabolism, loss of trophic support, and alteration of epigenetic mechanisms. Amyloid plaques and tau can impair synaptic functions by interfering with neurotransmitter release, receptor trafficking, synaptic vesicle recycling, and synaptic gene expression. They can also induce synaptic toxicity by triggering oxidative stress, calcium deregulation, mitochondrial dysfunction, and apoptotic pathways [55]. The synaptic dysfunction involves the loss of synaptic vesicles, impairment of synaptic plasticity, and synaptic signaling disruption. These changes affect the brain’s capability to process and store information, leading to the symptoms of AD. It is not uniform across brain regions and circuits but rather affects the hippocampus, the entorhinal cortex, and the neocortex, which are involved in memory formation and recovery. It is closely correlated with cognitive decline and dementia and, therefore, represents a potential target for therapeutic intervention [56]. Synaptic dysfunction is a promising therapeutic strategy that aims to restore synaptic function and plasticity and prevent or reverse synaptic dysfunctions in AD. For instance, modifying the disease process by reducing the levels or effects of amyloid-β and tau, the main proteins aggregate that accumulate in the patient’s brain and impair synaptic function. Targeting the intermediate mechanisms like inflammation, oxidative stress, mitochondrial dysfunction, and impaired neurotransmission, which mediate synaptic dysfunctions. Enhancing the synaptic functions and plasticity directly by modulating synaptic receptors, signaling pathways, or synaptic proteins. Pipenemab is an antibody that blocks the activity of semaphorin 4D, a protein responsible for the inhibition of synaptic growth and function [57].

### 3.5 Mechanism of Action

#### 3.5.1 Amyloid-β Plaques

**Fig. (5):**
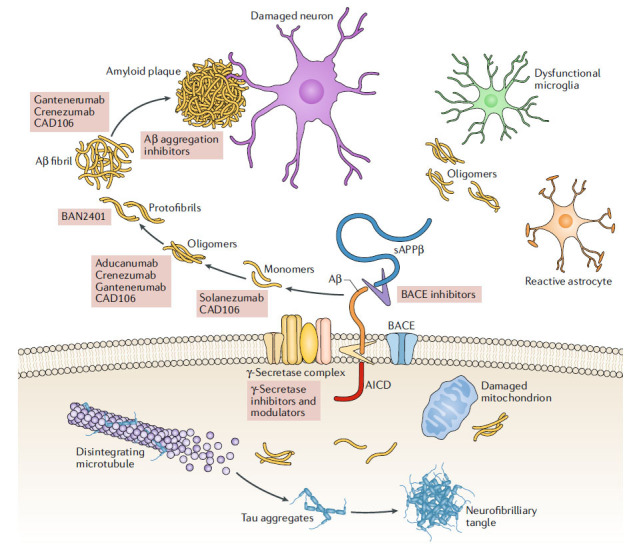
Generation mechanism of amyloid-β plaques and possible drug targets [17]. Copyrights^®^ Nature 2019.

Amyloid-β plaques are a prominent pathological feature in AD, making them a principal focus of therapeutic intervention (Fig. **5**). However, the specific mechanisms of action of various anti-Aβ strategies may vary depending on the type, stage, and location of Aβ aggregates. Some possible mechanisms of action for targeting Aβ plaques include:

1. Antibodies such as Abp3-42 can bind to deposited Aβ plaques, facilitating their clearance by microglia or other mechanisms. This may reduce the toxic effects of Aβ plaques on surrounding neurons and synapses [57].
2. Antibodies such as BACE inhibitors block the cleavage of amyloid-β protein precursor (AβPP) by β-secretase, hence lowering the formation of new Aβ plaques. This treatment may reduce the amounts of both soluble and insoluble amyloid-β and the development of plaque from it [57].
3. Compounds like NMDA receptor antagonists can protect neurons from excitotoxicity induced by the interaction of Aβ plaques with NMDA receptors. Blocking this toxicity prevents the loss of synaptic function and neuronal death caused by excessive calcium influx [58].
4. Reagents, like antioxidants, can modify factors that influence the formation, stability, and toxicity of Aβ plaques. This modification may alter the properties and effects of plaques on the brain [59].

#### 3.5.2 Tau Proteins

Tau proteins are involved in stabilizing the microtubules that support the structure and function of neurons. In AD, tau proteins become unnaturally phosphorylated, diminishing their affinity for microtubules and causing them to aggregate into neurofibrillary tangles. These tangles impair the communication between cells, leading to cell death. Thus, targeting tau proteins in this disease is a promising therapeutic approach aimed at preventing or reversing the formation of tangles and restoring neural functions. There are different strategies for targeting tau proteins:

##### 3.5.2.1 Immunotherapy

**Fig. (6):**
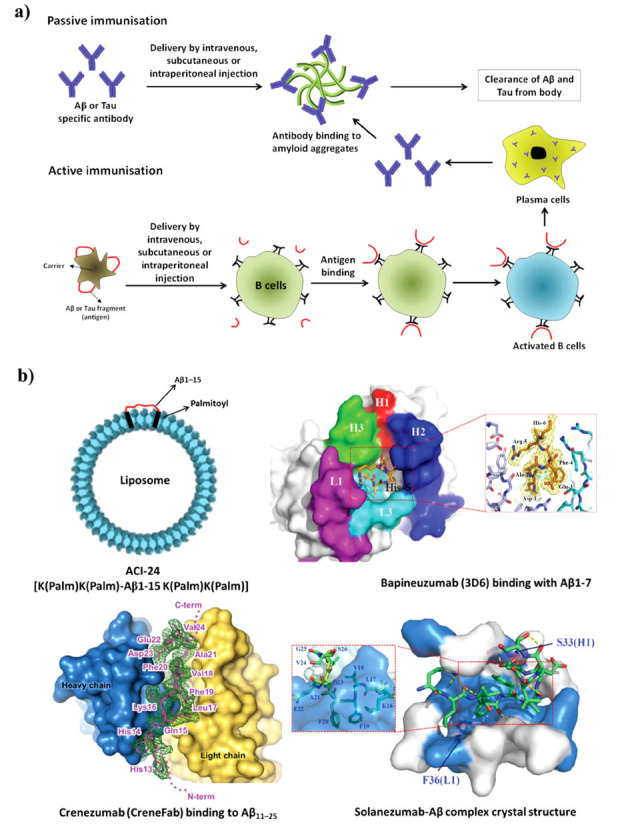
(**a**) Active and passive immunotherapy. (**b**) Liposome-based active immunization and interaction of anti-Aβ antibodies with Aβ. This figure has been adapted from [62-64].

Immunotherapy is a strategy that employs antibodies that bind to tau and remove it from the brain, either by activating the immune system or breaching the blood-brain barrier. Immunotherapy exists in both active and passive forms, depending on whether the antibodies are produced by the patient’s immune system or administered externally [60]. Immunotherapy for AD is often divided into two categories: (i) passive immunization, in which patients obtain antibodies against Aβ or tau, and (ii) active immunization, in which patients receive tau or Aβ as an antigen (Fig. **6**). Antibodies bind to plasma Aβ and promote its elimination from the bloodstream, ultimately leading to a gradient effect and the removal of Aβ from the brain. This mechanism is supposed to clear the brain of amyloid plaques. Conversely, antibodies have the ability to attach themselves to Aβ or tau tangles and trigger macrophages, thereby influencing the brain's ability to eliminate Aβ or tau clumps [22]. Nowadays, immunotherapy has become much more important than previous treatment approaches; specifically, it has been discovered that targeting amyloid aggregates (Aβ and tau) is a viable strategy. Prominent pharmaceutical corporations have recognized the significance of the amyloid hypothesis and amyloid aggregates as pharmacological targets, facilitating the development of medicines that affect the pathophysiology of AD [61].

##### 3.5.2.2 Stabilizing Microtubules

Stabilizing microtubules through drugs enhances the interaction between microtubules and tau, thus preventing tau from detaching and aggregating. Some examples of such drugs are paclitaxel, epothilone D, and davunetide [65].

##### 3.5.2.3 Kinase Inhibitors

These drugs inhibit the kinase enzyme responsible for phosphorylating tau protein, reducing the levels of hyperphosphorylated tau and its aggregation. Examples of such drugs include lithium, sodium selenate, and tideglusib [66].

#### 3.5.3 Neuroinflammation

Neuroinflammation refers to the activation of microglia and astrocytes, resulting in the production of inflammatory cytokines and the modulation of synaptic functions and neural survival. Targeting neuroinflammation may have beneficial effects in reducing disease progression and severity.

##### 3.5.3.1 Inhibition of NLRP3 Inflammasome

The NLRP3 inflammasome is a multiprotein complex that senses various stimuli, such as Aβ plaques and tau and activates caspase-1, which cleaves the pro-inflammatory cytokines IL-1β and IL-18. Inhibiting this may prevent the activation of microglia and astrocytes, reduce the production of inflammatory cytokines, and attenuate the neurotoxicity of Aβ plaques and tau [67].

##### 3.5.3.2 Modulation of Microglial Phenotypes

Microglia can adopt different phenotypes depending on environmental signals, such as M1 (pro-inflammatory) and M2 (anti-inflammatory). Modulating microglial phenotypes may shift the balance from a detrimental to a protective state, enhancing Aβ and tau clearance, promoting neurogenesis and synaptic plasticity, and suppressing inflammation [68].

##### 3.5.3.3 Targeting Astrocytes

Astrocytes, the most abundant glial cells in the brain, play important roles in maintaining extracellular homeostasis, supporting neuronal functions, and regulating the blood-brain barrier. Targeting them may regulate their interactions with neurons and microglia, reduce the release of inflammatory mediators, and improve Aβ and tau clearance [69].

##### 3.5.3.4 Interfering with Inflammatory Signaling Pathways

Several inflammatory signaling pathways, such as NF-κB, JAK/STAT, and MAPK, are involved in regulating gene expression, cytokine production, and cell survival. Interfering with these pathways may inhibit glial cell activation, suppress inflammatory gene expression, and protect neurons from apoptosis [70].

#### 3.5.4 Synaptic Dysfunction

Synaptic dysfunction occurs when the communication between neurons is disrupted by the accumulation of amyloid-β plaques. These plaques interfere with the synaptic plasticity, neurotransmission, and receptor functions. Targeting synapses is a potential strategy aiming to restore neuronal functions and prevent further damage.

##### 3.5.4.1 Enhancing Synaptic Plasticity

Enhancing synaptic plasticity involves the use of drugs or interventions that increase the ability of synapses to strengthen or weaken in response to neural activity. Examples include neurotrophic factors, cyclic AMP response element-binding proteins (CREB) activators, histone deacetylase (HDAC) inhibitors, and exercise [71].

##### 3.5.4.2 Protecting Synaptic Receptors

Utilizing medications or therapies that stop or fix the loss or malfunction of synaptic receptors, including metabotropic glutamate receptors (mGluRs), α-amino-3-hydroxy-5-methyl-4-isoxazolepropionic acid (AMPA) receptors, NMDA receptors, and others necessary for memory and learning. Examples of such drugs are memantine, ampakines, and mGluR antagonists [72].

##### 3.5.4.3 Reducing the Synaptic Toxicity

Synaptic toxicity can be mitigated by using drugs that decrease the production or aggregation of Aβ peptides or block their toxic effects on synapses. Examples of such strategies include immunotherapy, β-secretase inhibitors, γ-secretase inhibitors, and anti-Aβ antibodies [73].

## 4 EFFICACY AND SAFETY CONSIDERATIONS

### 4.1 Amyloid-β Plaques

Targeting Aβ plaques is a promising strategy to slow down or prevent the disease progression. Immunotherapy involves the use of antibodies that bind to plaques and clears them from the brain. Various preclinical and clinical studies on different types of anti-Aβ immunotherapy, such as active vaccines, passive monoclonal antibodies, and bispecific antibodies [74]. Some of the recent and notable studies are: aducanumab, a human monoclonal antibody that targets Aβ aggregates, was approved by the US Food and Drug administration (FDA) in 2021 under the accelerated approval pathway, based on its capability of reducing brain Aβ levels in patients with mild cognitive impairment. However, its clinical benefits remain uncertain due to conflicting results from two phase-3 trials assessing its impact on cognitive decline. The FDA has required a post-approval confirmatory trial to verify the clinical efficacy of the drug [39]. Lecanemab, another human monoclonal antibody that also targets Aβ aggregates, received accelerated FDA approval in 2023 based on its capability to reduce the Aβ levels in the brain. In a phase-2 trial, lecanemab showed a modest but statistically significant slowing of cognitive decline. An ongoing phase-3 trial aims to confirm its clinical efficacy [75]. UB-311, a synthetic peptide vaccine that induces antibodies against Aβ, demonstrated a favorable safety profile and robust immune response in phase-2 trials. While it showed trends toward cognitive improvement and disease stabilization, the results were not statistically significant. A phase-3 trial is planned to further evaluate its efficacy [39].

The main safety concern and side effects associated with anti-Aβ therapies are amyloid-related imaging abnormalities (ARIA), which are swelling of the brain (edema) or bleeding (hemorrhage) caused by the removal of plaques and are detected through MRI. These abnormalities can cause headache, confusion, dizziness, nausea, seizure, and other neurological symptoms and may require dose adjustment or treatment discontinuation. Such abnormalities are more common in patients who carry the *APOE4* gene, which is a risk factor for the disease. Other possible side effects include injection site reaction, infection, allergic reaction, and inflammation [76].

### 4.2 Tau Proteins

As tau proteins are involved in the formation of neurofibrillary tangles in AD, targeting them is a promising strategy to reduce tau pathology and improve cognitive functions in patients. However, there are multiple challenges and uncertainties in the approach, such as the optimal target epitope, the best antibody format, the safety and tolerability of the treatment, and the biomarkers to monitor the efficacy and response [77]. A review of literature showed that several preclinical and clinical studies have been conducted to evaluate the effects of different anti-tau antibodies in disease treatment. A phase-2 study of semorinemab (NCT03828747), a humanized monoclonal antibody that binds to the mid-region of tau, showed no significant difference in cognitive decline between the placebo and treatment after 49 weeks. However, a subgroup analysis suggested it might have some benefit in patients with mild AD and higher tau levels [78]. Similarly, another phase-2 study of a humanized monoclonal antibody, gosuranemab, which targets the N terminal region of tau, also failed to meet its primary endpoint of slowing the cognitive decline after 52 weeks. However, the study found some evidence of reducing tau pathology and neurodegeneration in the treatment group when measured by PET imaging and CSF biomarkers [78, 79]. Furthermore, a phase-2 study of humanized monoclonal antibody RO7105705, which binds to the C terminal region of tau, reported that the treatment group showed rapid and robust amyloid plaque clearances and changes in relevant downstream biomarkers among patients with prodromal or mild to moderate AD. Its primary outcome is the change in cognitive functions as assessed by the ADAS-Cog13 scale. At week 25 of treatment, patients demonstrated decreases in CSF total tau, CSF p-tau181, and CSF neurogranin. As per the latest report (December 2024), a Phase Ib/IIa, randomized, double-blind, placebo-controlled study is recruiting to investigate the safety, tolerability, pharmacokinetics, and pharmacodynamics of RO7126209 following intravenous infusion in patients with mild to moderate AD [79]. A phase-1 study of monoclonal antibody ABBV-8E12, which recognizes the N terminal region of tau, showed a favorable safety and tolerability profile in patients with progressive supranuclear palsy (PSP), a tauopathy that shares some features with AD. The study reported a reduction in tau accumulation and brain atrophy in the treatment group, as well as a trend toward slower clinical decline [80]. Another phase-1 study of BIIB092, a humanized monoclonal antibody that targets the microtubules-binding region of tau, reported a dose-dependent reduction in CSF tau levels in patients with mild to moderate AD. The study also showed a trend toward lower tau PET signal and less brain volume loss [81].

Some of the safety concerns and adverse events related to anti-tau immunotherapies have been reported. Infusion-related reactions are the common side effects of IV administration of monoclonal antibodies. It may include headache, nausea, fever, rash, or anaphylaxis. Brain edema or microhemorrhage can occur after anti-tau therapy, which can cause neurological symptoms like headache, confusion, and seizures. Cerebral microbleeds may also occur, which are small hemorrhages in the brain when detected through MRI. These are associated with an increased risk of stroke, cognitive impairment, and dementia. Some anti-tau antibodies may increase their risk by affecting the BBB or coagulation system [79]. Psychiatric problems may also appear, which are behavioral and emotional disturbances. Some anti-tau antibodies, such as RO7105705, may induce psychiatric symptoms, such as anxiety, depression, psychosis, or agitation [82].

### 4.3 Neuroinflammation

Several preclinical and clinical studies have reported various anti-inflammatory agents, such as NSAIDs, cytokine inhibitors, immunomodulators, and antioxidants, for their efficacy in AD. A review article by Chen and Yu discusses the interplay mechanisms and clinical translation of tau and neuroinflammation in AD. They summarize the latest evidence on current clinical trials targeting tau and neuroinflammation [78]. Another study highlights the translational evidence for various anti-inflammatory strategies like anti-inflammatory molecules, nutraceuticals, endocannabinoid systems, and gut microbiota. They highlighted the importance of a broad multimodal approach for treating neuroinflammation [83]. Another study aimed to determine whether neuroinflammation, measured by PET imaging, is associated with Aβ plaques, cognitive decline, or plasma biomarkers of inflammation. They also investigated the effects of anti-inflammatory drugs on neuroinflammation and cognition in AD patients [84]. A study by Chen *et al*. examined the role of neuroinflammation as a contributing factor for AD and potential therapeutic targets and strategies to regulate neuroinflammatory pathways [78]. Furthermore, a study by El Idrissi *et al*. used computational approaches for exploring the basic molecular network in neuroinflammation. They reported 11 key proteins that are most likely modulating the neuroinflammation and suggested possible drug candidates and combinations for targeting them. However, these studies are inconclusive and mixed because different agents may have different mechanisms of action, pharmacokinetics, and safety profiles [85].

### 4.4 Synaptic Dysfunction

Several studies related to targeting synaptic dysfunction in recent times have been conducted, a brief overview is explained here. A preclinical study reported the effects of enhancing tripartite synapses on synaptic function and cognition in a mouse model of tauopathy (rTg4510). The study investigated an increasing expression of CD38 protein in astrocytes, which improves synaptic transmission, long-term potentiation, and memory performance in mice [86]. Another preclinical study examining the role of synaptic dysfunction in the generation and propagation of abnormal oscillatory activity in the hippocampus of mice (APP/PS1). The study reported an impaired balance between excitation and inhibition in the hippocampus network, resulting in theta and gamma oscillations and decreased sharp wave ripples. It also examined that reducing synaptic dysfunction with rapamycin normalized oscillatory activity and improved spatial memory in mice [87]. Another study reported the effects of rolipram (1.25 mg/kg for 24 days) on memory deficits in APP/PS1/tau triple transgenic mice. Rolipram reduced tau phosphorylation, increased neuronal survival, normalized glial cell function, and inhibited cognitive decline by downregulating amyloid-β, amyloid precursor protein, and presenilin 1. In order to decrease apoptosis, rolipram additionally downregulated Bcl-2-associated X protein (Bax) and raised B-cell lymphoma-2 (Bcl-2); interleukin-1β, interleukin-6, and tumour necrosis factor-α to limit neuroinflammation. Furthermore, rolipram decreased EPAC1 and elevated cAMP, PKA, 26S proteasome, EPAC2, and phosphorylation of ERK1/2 [88].

Some of the safety issues and adverse effects that have been reported in preclinical and clinical studies of synaptic therapies are:

1. Infusion related reactions are the common side effects of IV administration of monoclonal antibodies. It may include headache, nausea, fever, rash, or anaphylaxis.
2. Amyloid-β related imaging abnormalities are brin edema or microhemorrhages that occur with amyloid and tau immunotherapy. This can cause neurological symptoms like headache, confusion, or seizure.
3. Cerebral microbleeds are small hemorrhages in the brain that can be detected by MRI. This may increase the risk of affecting BBB and the coagulation system.
4. Behavioral or emotional disturbances that affect the mood, cognition, or personality of patients [89].

### 4.5 Emerging Research Efforts and Future Implications

Targeting amyloid-β plaques as novel drug targets in AD is promising, as they may offer more potent and safer alternatives to the current therapies. Several experimental therapies have been designed to reduce the Aβ plaques in the brain, either by inhibiting the enzyme that produces Aβ from its precursor protein or by enhancing the clearance of Aβ through immunization or passive antibody administration. Numerous novel drugs are being developed to selectively bind and neutralize Aβ oligomers, such as ACU193, an antibody that has shown positive results in a phase-1 trial and is expected to enter phase-2 in 2024. In addition, numerous drugs with different mechanisms of action and targeting sites are going through the clinical trial phases (Table **1**) [90]. Targeting tau as a novel drug site is promising to slow down or prevent the progression of the disease. Currently, there are four clinical candidates in phase-3 trials and 16 in phase-2 trials for targeting tau therapies. Phase 3 trials are being conducted on azeliragon, a small drug that binds to the receptor for advanced glycation end products (RAGE) for the treatment of moderate AD [91]. LMTX is a small molecule that inhibits the aggregation of tau and amyloid-β and is in phase-3 trials for mild to moderate AD. Semorinemab, a monoclonal antibody, binds to the N-terminal region of tau and prevents its aggregation and propagation, and is in phase-3 trials for prodromal to mild AD. Gosuranemab, a monoclonal antibody that binds to the C-terminal region of tau and blocks its seeding and spreading, is in phase-3 trials for mild AD [6].

Several investigational drugs that target neuroinflammation in AD are currently in clinical trials. Donanemab and lecanemab monoclonal antibodies that bind to Aβ plaques and also reduce neuroinflammation triggered by Aβ. Both drugs have shown positive results in phase-3 trials, and lecanemab has received accelerated approval by the FDA in 2023 [92]. Naproxen and celecoxib NSAIDs inhibit the enzyme cyclooxygenase (COX 2), which is involved in the synthesis of prostaglandins. These drugs may prevent or delay the onset of AD by reducing the chronic inflammation in the brain [92]. Two anti-inflammatory antibiotics, minocycline and doxycycline, have the ability to prevent neurons from being harmed by cytokines and nitric oxide produced by microglia and astrocytes [93]. Resveratrol and curcumin are two naturally occurring substances with anti-inflammatory and antioxidant qualities. Free radicals and reactive oxygen species, which are produced by neuroinflammation and result in oxidative stress and neural damage, can be scavenged by them [94]. During recent years, the novel drug targeting site synaptic dysfunction has been a hopeful research direction. Synaptic biomarkers, such as synaptic vesicle proteins, neurogranin, and synaptotagmin, can be measured in cerebrospinal fluid or by positron emission tomography imaging. These biomarkers can be used for synaptic therapy efficacy assessment, disease diagnosis, and disease progression monitoring. Compared to alternative strategies, these therapies may have a higher chance of changing the course of the disease and may affect cognitive function more directly and immediately. More research is required to confirm the safety and efficacy of these therapies in humans, some of which have demonstrated promising results in animal models and clinical trials [95].

## 5 TRANSLATIONAL INFORMATICS FOR DIAGNOSIS AND MANAGEMENT OF AD

According to the American Medical Informatics Association (AMIA), translational informatics (TI) refers to the process of developing storage, analytical, and interpretive methods to maximize the conversion of genomic and biomedical data into proactive, predictive, preventive, and participatory health [105]. TI is an emerging area of bioinformatics research that explores its clinical uses. It results from the convergence of artificial intelligence-based technologies, big molecular data, omics data, biostatistics, and big data in the health care industry. Building disease models, developing drug designs, and predicting diseases are all made possible by bioinformatics knowledge bases and tools. To interpret clinical data, TI uses methods from data mining and data analysis. It is a multidisciplinary approach that uses computer-assisted building techniques to develop information systems that enhance medical care. The integration of artificial intelligence-based technologies has brought a lot of attention to this field in recent years. A promising method for obtaining quick and accurate results from laboratory experiments is the synergistic integration of data with clinical data [106].

With recent advances in a variety of digital technologies, including wearables, discrete sensors, and passive data collection applications, combined with improved computational power and analytical methods, a more complex and comprehensive understanding of the onset and progression of AD and other neurodegenerative diseases appears possible. Digital technology may potentially improve diagnosis sensitivity and specificity, as well as the ability to choose relevant trial participants, detect small changes in cognition and function across the illness spectrum, and analyze therapeutic responses more accurately. Digital technology could potentially improve patient care and safety by encouraging age-in-place therapy and allowing the development of precision medicine treatment strategies [107]. The National Institute on Aging finances Alzheimer's disease centers (ADCs), and the National Alzheimer's Coordinating Center (NACC) is in charge of creating and maintaining a database including patient information from these ADCs. After patients are enrolled, each center gathers data elements that are specific to that center and sends a minimum dataset to NACC. Depending on the needs of each center's research, data are managed differently at each center. The data systems used by the centers range from a single workstation with spreadsheet software to a network of servers with an advanced data management system, like Oracle [108]. Wearable accelerometers worn on the ankle have been used to monitor changes in daily mobility behavior in AD patients, even when no significant behavioral abnormalities exist. High-frequency, in-home monitoring data has proven to differentiate between people with moderate cognitive impairment and those with normal cognitive function [109]. This implies that the information may reduce the sample sizes needed for clinical studies, lowering the number of individuals exposed to potentially harmful medications. When assessing personalized/precision medicine techniques in clinical trials, digital technologies with continuous measurements may also make it possible to identify unique behavioral profiles' sensitivity to placebo reactions or to use run-in data to lessen regression to the mean effects. An augmented reality paradigm was used in the test produced by Altoida Inc. Preliminary evidence indicates that this test may identify, six years ahead of time, whether a person would acquire AD dementia with a 94% accuracy rate. The Altoida Neuro Motor Index (NMI) is a 10-minute test that employs an iPhone app with changing difficulty levels to examine psychomotor processing speed, mental agility, short-term memory, and visual recall. Although a combination of AD biological markers (fluorodeoxyglucose-positron emission tomography, magnetic resonance imaging, electroencephalography/event-related potentials, and phosphorylated tau protein/amyloid-β 42 in the cerebrospinal fluid) outperforms NMI alone, NMI is a better predictor of AD due to its correlation with other biomarkers [110, 111].

### 5.1 Ontologies

The amount and complexity of data acquired in the biological sciences, as well as the opportunities for analyzing this data, have undergone a dramatic revolution in the previous few decades. These data are extensively spread and highly heterogeneous due to their scattering, variety of forms, and generation by various technologies [112]. In order to enhance patients' quality of life, research activities must be successful, and one key objective is to facilitate the development of an appropriate infrastructure for the standardization, interchange, and sharing of information. Given that, the emergence of ontologies has been shown to be among the best answers in the biomedical field, particularly in neuroscience, where mental processes are explained at several levels of abstraction [113]. Within a particular field of knowledge, an ontology is a formal specification of classes, attributes, and their connections. This promotes the integration and recovery of heterogeneous data from many sources, which enhances the diagnosis and treatment of various diseases, including AD. It also enables standardization and clarity in the representation of a domain, which improves information transmission [114].

The Semantic Web Applications in Neuromedicine (SWAN) project by Gao *et al*. (2006) produced one of the first ontologies designed to store and contextualize pre-existing AD data. The authors claim that SWAN offers a uniform standard, and both doctors as well as researchers could use it. In order to facilitate the creation of a semantic network of theories, publications, and digital archives at the time, the project was designed as an infrastructure that successfully integrated the body of scientific information already known about AD [115]. This ontology, along with its accompanying application, was formerly the web's reference source for AD knowledge, but it has since been withdrawn from all repositories where it was kept. Each component of the AD domain is to be comprehensively covered in an organized manner by the Alzheimer's Disease Ontology (ADO). Including information on diagnosis, therapy, and biological causes, this is one of the most thorough ontologies. ADO is notable for its comprehensive covering of cognitive processes despite its restricted scope in certain subdomains [112]. Similar to SWAN, ADO was developed to make it possible for stored data to be retrieved and interpreted *via* queries. Nevertheless, it is challenging to comprehend the axiomatic framework [116]. The Neurodegenerative Disease Data Ontology (NDDO) is an ontology that attempts to capture information on neurodegenerative disorders, namely Parkinson's and AD. To enable reasoners to deduce new information from facts, it aims to simplify the process of conceptually annotating data on diagnosis and illness development. Because NDDO has a high degree of term reuse, other research groups can benefit from improved interoperability and reusability [117]. Research, diagnosis, therapy, and care are all improved in different ways by employing structured knowledge representations in ontology-based AD management. Ontologies significantly contribute to the organization and sharing of domain-specific knowledge about AD [118].

### 5.2 Analytics

In the context of AD, analytics play a pivotal role in enhancing research, diagnosis, therapy, and care through the use of predictive analytics, explainable AI, and data-driven approaches. Predictive analytics, in particular, is being used in the management of AD to enhance early diagnosis and care. Predictive analytics may help with diagnosis and risk factor identification by analyzing data from several sources, such as biomarkers and clinical data [119]. To improve early disease detection, a deep learning-based classification model with an integrated feature selection technique was utilized to identify AD patients. The study used an AD DNA methylation data set (64 records, 34 patients, and 34 controls) from the GEO omnibus database. Before selecting important features, the data underwent downstream analysis, quality checks, and normalization. Four embedded-based feature selection methods were examined since there were a great number of related CpG sites; the optimal technique was then applied to the suggested classification model. Other categorization models included a Convolutional Neural Network (CNN), a Recurrent Neural Network (RNN), and a Deep Recurrent Neural Network (DRNN), which were compared to an Enhanced Deep Recurrent Neural Network (EDRNN) [120].

### 5.3 Explainable AI

Explainable AI models have been constructed employing multimodal datasets, including clinical, MRI segmentation, and psychological data, for predicting the onset and course of AD. For instance, a recent study described a machine learning prediction model for identifying prodromal AD patients in the general population. The authors set out to construct a machine-learning prediction model to help primary care physicians identify AD early and refer patients to expert locations for biomarker confirmation and clinical trial registration. According to the study findings, the machine learning algorithm helps identify prodromal AD individuals in the general population who have yet to be identified [121]. Furthermore, a preventative and predictive strategy was developed to identify, predict, and prevent the onset of AD using biomarkers such as the amyloid-β protein. A model based on Convolution Neural Networks (CNNs) was developed to forecast AD in its first phases. The outcomes demonstrated how effectively the suggested model works in comparison to conventional machine learning (ML) techniques, including K Nearest Neighbor, Decision Tree Classifier, Support Vector Machine, and Logistic Regression [122]. Moreover, a machine learning model based on GradientBoost, XGBoost, Decision Tree, Random Forest, GaussianNB, and Voting Classifier was created to predict AD. Accuracy, precision, recall, and F1 score were evaluated for performance using the open access series of imaging studies (OASIS) dataset, which was utilized to train the model. According to the findings, for the AD dataset, the voting classifier had the highest validation accuracy of 96%. In light of this, ML algorithms may significantly reduce the annual death rates from AD by accurately identifying the disease at early stages [123]. A recent study employed a machine learning system to detect AD. The performance of the prediction model, which was built using data from the Open Access Series of Imaging Studies (OASIS), was evaluated using measures such as F1-score, precision, recall, and accuracy. The proposed research produced better results, with the highest validation average accuracy of 83% on AD test data. Compared to previous studies, this test's accuracy score is considerably greater [124].

### 5.4 Data Fusion and Multimodal Approaches

Data fusion approaches, which integrate several data sources, including clinical, imaging, and psychological data, are used in analytics for managing AD. The ultimate goal is to enhance prediction accuracy and give a full picture of an illness such as AD. For instance, a recent study presented a novel multimodal data fusion framework that uses dynamic FC (dFC)-based fMRI feature extraction and deep residual learning of non-linear sMRI features to predict the subset of people with mild cognitive impairments who will develop AD within three years of baseline scanning sessions. Cross-validated results from the developed multimodal (sMRI-fMRI) data fusion framework showed a significant improvement in performance over unimodal prediction studies employing the fMRI (p = 7.03 x 10^-7^) and sMRI (p = 6.72 x 10^-4^) modalities [125]. Recently, another unique multimodal data fusion technique was proposed, along with a new machine learning framework that includes feature selection, data fusion, classification, and the extraction of disease-causing factors. The framework's efficiency was proven using an actual dataset of 37 AD patients and 35 normal controls (NC) with functional magnetic resonance imaging (fMRI) and genetic data. It outperformed other methods in classification and optimal feature extraction. In the meanwhile, genes and brain areas associated with AD have been identified, including the olfactory cortex, insula, posterior cingulate gyrus, lingual gyrus, CNTNAP2, LRP1B, FRMD4A, and DAB1. According to the results, the machine learning framework successfully performed multimodal data fusion analysis, creating new opportunities for AD detection [126]. Based on longitudinal 3D MRI volumes gathered over a period of six months, a study was carried out that looked at the impact of combining cross-sectional biomarkers with MRI, including the demographic and cognitive scores of the patients from the initial visit. Furthermore, the authors introduced a new explainability methodology that facilitates the comprehension of the final result of the suggested multimodal approach by practitioners and domain experts. The results of extensive trials indicate that the suggested framework has 96%, 99%, 92%, and 96% accuracy, precision, recall, and area under the receiver operating characteristic curve, respectively [127].

The idea of predicting the incidence of dementia across all nations in the globe using a collection of characteristics related to personal health, food intake, substance use, and abuse, and the effectiveness of the healthcare system is being investigated using artificial intelligence and complex systems physics. The analysis refers to a 26-year time span and employed a publicly accessible indicator variable at the national level. Using techniques grounded on explainable Artificial Intelligence (XAI) and complex networks, the researchers identified a set of lifestyle factors mostly related to nutrition that have the greatest impact on predicting the prevalence of dementia and AD [128]. A paradigm for the precise and understandable diagnosis and progressive detection of AD was given in an alternate study. The model contains 11 modalities of 1048 patients from the Alzheimer's Disease Neuroimaging Initiative (ADNI) real-world dataset, including 294 cognitively normal, 254 stable, moderate cognitive impairment (MCI), 232 progressing MCI, and 268 AD. Random Forest (RF) is the classifier method used in the two-layer model. To help in the early identification of AD patients, the model does a multi-class classification in the first layer. The model's second layer uses binary classification to identify potential MCI-to-AD progression within three years of a baseline diagnosis. Critical factors that improve the model's performance are chosen from a diverse set of biological and clinical variables. To provide global and instance-based explanations of the RF classifier for each layer, the researchers used the SHapley Additive ExPlanations (SHAP) feature attribution framework. Furthermore, they developed 22 explainers based on fuzzy rule-based systems and decision trees in order to provide further justifications for every RF option at every tier. The created model obtained an F1-score of 87.09% and a cross-validation accuracy of 87.08% in the second layer, while it earned an F1-score of 93.95% and an F1-score of 93.94% in the first layer. Because of the explanations provided, which are typically consistent with each other and the AD medical literature, the resulting procedure is responsible, dependable, safe, and appropriate for use in medicine. The proposed method can help advance clinical understanding of AD diagnosis and progression processes by providing thorough insights into the influence of several modalities on the disease risk [129].

Using the nine most popular machine learning algorithms, five class groupings are predicted. This category includes the following models: Random Forest (RF), Adaptive Boosting (AdaB), Gradient Boosting (GB), Decision Tree (DT), Logistic Regression (LR), Support Vector Machine (SVM), K-Nearest Neighbor (KNN), and Naive Bayes. RF has received the highest rating among all of these models. This study employs SHapley Additive Explanation (SHAP) in addition to explainability. The RF classifier's performance review demonstrates that it can detect AD, non-Alzheimer's dementia, cognitively normal, unexplained dementia, and other illnesses with a 10-fold cross-validation accuracy of 98.81%. Additionally, this work used Explainable Artificial Intelligence, which is based on the SHAP paradigm, to investigate the causes of prediction. Using data from MRI segmentation, clinical psychology, and the Open Access Series of Imaging Studies (OASIS-3) dataset, this study presented a multimodal five-class classification of AD [130]. A recent study found that the course of AD may be predicted using a hybrid CNN-LSTM-based model that integrates four longitudinal cognitive sub-score modalities. The hybrid model improves the process of selecting the best architecture for the deep learning model by using the Bayesian optimizer as a computational technique. Instead of using the traditional SoftMax classifier, a robust and efficient random forest classifier has been employed to choose the best feature set from the CNN-LSTM's recovered deep representations. As an additional optimization stage, a feature selection process based on genetic algorithms has been included. A thorough set of experiments utilizing the ADNI dataset examined the usefulness of each optimization step and demonstrated the effectiveness of the hybrid model that was recommended. In terms of results and performance, the model was particularly better than other deep learning and conventional machine learning approaches. To ensure diagnostic interpretability, the researchers used the SHAP and LIME techniques to provide explainability features for the decisions in the proposed model [131].

## 6 CHALLENGES AND FUTURE PROSPECTS

While there has been significant progress in developing therapeutic approaches to prevent AD symptoms, numerous drug candidates have sadly failed at different stages of clinical trials [132]. The primary reason for these shortcomings is that the majority of these drugs block one of the several disease pathways associated with multifactorial AD. Put another way, a good therapeutic design must include the integration of numerous disease pathways since the complicated pathophysiology of AD involves the malfunctioning of multiple important biochemical processes. In this regard, the multifunctional approach used in laboratories may prove to be a fruitful and efficient method for creating medications that specifically address the complex pathophysiology of AD [133]. In most cases, drugs that target inflammation and the cholinergic system just relieve symptoms. However, when combined with multifunctional medications that address the underlying pathology of AD, these medications can become more effective and have better therapeutic results [134]. Despite the abundance of published research, our knowledge of AD's intricate molecular and pathophysiological pathways is still somewhat restricted. Actually, it is necessary to identify and confirm the true cause and feasible molecular targets using both new and old information [29].

Various approaches, including small compounds, natural products, peptidomimetics, and metal chelators, have been used to combat amyloid aggregation (Aβ and tau); however, the disappointing results have redirected researchers' attention toward the development of multifunctional drug candidates [135]. Such therapeutic candidates ought to be able to halt a wide range of activities, including inflammation, mitochondrial damage, synaptic toxicity, excessive ROS production and oxidative stress, metal chelation, metal-induced aggregation, and damage to biomolecules caused by ROS. Leading pharmaceutical companies have been aggressively pursuing immunotherapy in recent years since it is seen to be one of the most likely therapeutic options for aiming to target the clearance of amyloid from the brain [136]. While immunotherapy is the focus, several important concerns need to be resolved before it can be employed in clinical applications. These issues include low blood-brain barrier permeability, size, autoimmune reaction, stability, retention in the brain, brain bleeding and shrinkage, and high development and treatment costs [137].

One promising approach to drug development in AD pathophysiology is to inhibit the activities of enzymes, including β-secretase, γ-secretase, and kinases. Due to their critical function in many other biological processes in the brain, current efforts targeting these enzymes have demonstrated unfavorable side effects. Thus, we suggest designing molecular clamps that attach to certain phosphorylation sites in Tau or the β-secretase/γ-secretase cleavage site in AβPP to stop the unwanted specific activity of enzymes. Potential drug candidates that efficiently block enzymatic (phosphorylation/protease) activity without interfering with the other biological activities of enzymes may be produced by using this therapeutic design technique [138]. Lastly, it is worth mentioning that AD is now becoming a widespread epidemic around the globe. New and faster ways to manage the illness through efficient therapeutic and diagnostic treatments must be provided by the scientific community across disciplinary boundaries. The Human Brain Project (HBP), the ambitious neuroscience and brain projects Brain Mapping by Integrated Neurotechnologies for Disease Studies (BRAIN/MINDS) in Japan, the China Brain Project in China, and the Brain Research through Advancing Innovative Neurotechnologies (BRAIN) Initiative in the United States are all endeavors to enhance our knowledge of and capacity to treat brain disorders, principally AD [139, 140].

To cut the long story short, there has been a significant shift from the traditional concepts about AD that correlate plaques and tangles to the overproduction of amyloid-β and hyperphosphorylated tau, respectively. Lipopolysaccharide causes elevated levels of toll-like receptor (TLR)-4 signaling, and endogenous ligands like high mobility group box 1 (HMGB1) activate local “alertin,” resulting in the alleviation of amyloid-β [141]. Alleviated levels of amyloid-β result in accumulation and have been reported to harm brain tissues [142]. Similar to amyloid-β, the elevated levels of hyperphosphorylated tau might be caused by the aging-induced decrease of local and pineal melatonin synthesis. The transcription factors NF-κB, and yin yang (YY)1 are stimulated when LPS/HMGB1 activates TLR-4, increasing the synthesis of BACE1 and amyloid-β. Both NF-κB and YY1 have the ability to enhance the melatonergic pathway [142-144]. Possibly through the activation of the NF-κB component, c-Rel, by the specialized pro-resolving mediator, neuroprotection D1 [144, 145]. After being activated by NF-κB-driven processes, c-Rel increases the induction of the melatonergic pathway in macrophages and microglia. Melatonin that is released has autocrine and paracrine effects that not only reduce inflammation but also stop tau hyperphosphorylation [146]. According to a recent meta-analysis, melatonin is more effective than aducanumab, lecanemab, and donanemab. This offers a different perspective on the pathophysiology of AD that takes into account the broader complexity of neurodegenerative changes occurring and offers a more nuanced focus for future treatments than the bombing of amyloid-β by anti-amyloid-β antibodies, which have negligible effect [147].

## CONCLUSION

After thoroughly reviewing the literature, we came across a lot of research articles that focused on predicting the onset of AD through translational informatics and identifying potential drug-targeting sites. Without a doubt, these studies have broadened our understanding of the management and treatment of AD. Specifically, the translational informatics approaches using AI and multimodal algorithms have possibly led to the early prediction of the disease with up to 96% accuracy. In addition, the current treatment for AD offers symptomatic relief but fails to address the disease progression. This has shifted the focus towards the discovery of novel drug sites that promise symptom alleviation as well as modification of disease course. Emerging drug sites have introduced a new approach to disease treatment, offering mechanisms of action that could potentially slow down or even stop the disease progression. We discussed more than 40 drugs along with possible mechanisms that are currently going through clinical trials. On the drug development front, significant progress has been made in identifying novel drug targets, including amyloid beta (Aβ) aggregation, tau phosphorylation, and inflammation. Drugs like Aducanumab, Lecanemab, and Gantenerumab, which aim to clear amyloid plaques, as well as tau aggregation inhibitors like LMTX, are currently under investigation for their clinical efficacy. However, challenges remain in the drug development process, particularly with drugs targeting β-secretase and γ-secretase, as they have shown limited success due to side effects. Ongoing research and clinical trials continue to develop our understanding of the disease and cover the way for innovative prediction algorithms, detection methods, and treatment modalities for AD. In conclusion, the significance of ongoing research and development in this area should not be overstated, as it has the potential to open up new options for prediction and therapy options and enhance the prognosis of patients with AD. We really hope that more studies in this field will lead to the development of new drugs, the achievement of earlier predictions, and real advantages for people who are struggling with AD.

## Figures and Tables

**Fig. (4):**
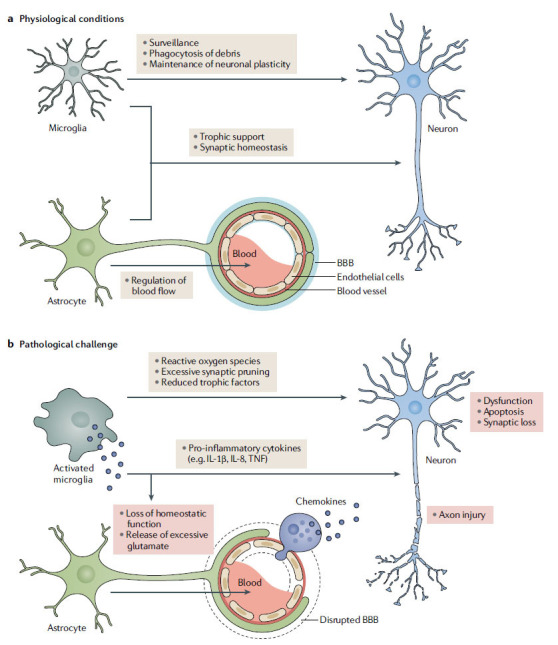
Molecular pathways and mediators involved in neuroinflammation during AD [53]. Copyright^®^ Nature 2021.

**Table 1:** List of drugs for AD, either approved by the FDA or currently going through the clinical trial phase.

**Drug**	**Targeting Site**	**Mechanism**	**Study Design (Trial Phase)**	**Trial Number**	**References**
Aducanumab	Amyloid-β	Antibodies targeting amyloid	FDA approved	BIIB037	[96]
Lecanemab	Amyloid-β	Antibodies targeting amyloid	FDA approved	BAN2401	[96]
ANAVEX^®^2-73 (Blarcamesine)	Amyloid-β	Targeting sigma-1 and M1 muscarinic receptors	Phase 2B/3	NCT02244541	[97]
Donanemab	Amyloid-β	A monoclonal antibody targeting deposited plaques	Phase-3	LY3002813	[98]
ACU193	Amyloid-β	An antibody targeting Aβ oligomers	Expected to enter phase-2 in 2024	NCT04931459	[99]
BIIB080 (IONIS-MAPTRx)	Tau proteins	Antisense oligonucleotide targeting the tau protein	Phase-1 with promising results	NCT05399888	-
Lithium	Tau proteins	GSK-3β inhibitor for MCI	Phase-4	NCT05423522	[66]
Azeliragon	Tau proteins	Bind to the receptor for advanced glycation and products (RAGE)	Phase-3	NCT03980730	-
Semorinemab	Tau proteins	Prevent aggregation and propagation of tau proteins	Phase-3	NCT02820896	-
Gosuranemab	Tau proteins	Prevent seeding and spreading of tau proteins	Phase-3	NCT03352557	[100]
CNP520	Synaptic dysfunction	Tau aggregation inhibitor	Phase-3	CNP520	[101]
Atabecetat (JNJ-54861911)	Synaptic dysfunction	Tau aggregation inhibitor	Phase-2	NCT04619420	-
BAN2401	Synaptic dysfunction	Tau aggregation inhibitor	Phase-3	NCT01230853	-
Sodium selenate	Tau proteins	PP2A activator	Phase-2	NCT01055392	[66]
LY3372689	Tau proteins	O-GlcNAcase inhibitor	Phase-2	NCT05063539	-
LMTM	Tau proteins	Aggregation inhibitor	Phase-3	NCT02245568	-
Curcumin	Tau proteins	Aggregation inhibitor	phase-2	NCT00099710	[102]
BIIB076	Tau proteins	Tau-targeting antibody	Phase-2	NCT03056729	-
Celecoxib	Neuroinflammation	Reduces the activation of microglia	Phase-3	NCT00065169	[103]
Pimavanserin	Neuroinflammation	Serotonin 2A receptor inverse agonist	Phase-3	NCT03118947	-
Pioglitazone	Neuroinflammation	Suppresses PGE2 synthesis inhibits PKA signaling triggered by EP2	Phase-2	NCT00982202	-
Neflamapimod (VX-745)	Neuroinflammation	Inhibit the p38 mitogen-activated protein kinase alpha (MAPKα) isoform	Phase-2	NCT03435861	[104]
Masitinib	Neuroinflammation	Inhibit mast cells and microglial activation	Phase-3	NCT05564169	-
NE3107	Neuroinflammation	Inhibit inflammatory pathways like ERK and NF-κB	Phase-3	NCT04669028	-
ALZT-OP1	Neuroinflammation	Inhibit cytokine release	Phase-3	NCT02547818	-
Dexmedetomidine	Synaptic dysfunction	Protect against cognitive impairment	Phase-1	NCT06052254	-
NP001	Neuroinflammation	Inhibit the activity of macrophages and microglial cells	Phase-2	NCT03179501	-
Pioglitazone	Synaptic dysfunction	Reverse memory impairment acting as a PPARγ agonist	Phase-2	NCT00982202	-

## LIST OF ABBREVIATIONS

AD: Alzheimer’s Disease
AI: Artificial Intelligence
AβPP: Amyloid-β Protein Precursor
CNNs: Convolution Neural Networks
DEPTAC: Dephosphorylating-targeting Chimera
fMRI: Functional Magnetic Resonance Imaging
GB: Gradient Boosting
HBP: Human Brain Project
ML: Machine Learning
NF-κB: Nuclear Factor Kappa B
NMDA: N-methyl-D-aspartate
NO: Nitric Oxide
PSP: Progressive Supranuclear Palsy
RNN: Recurrent Neural Network
SPECT: Single Photon Emission Computed Tomography
SVM: Support Vector Machine
TI: Translational Informatics
TLR: Toll-like Receptor
TNF: Tumor Necrosis Factors

## References

[1] Atri A. (2019). The Alzheimer’s disease clinical spectrum: Diagnosis and management.. Med. Clin. North Am..

[2] Thies W., Bleiler L. (2013). 2013 Alzheimer’s disease facts and figures.. Alzheimers Dement..

[3] Lanctôt K.L., Hahn-Pedersen J.H., Eichinger C.S., Freeman C., Clark A., Tarazona L.R.S., Cummings J. (2024). Burden of illness in people with Alzheimer’s disease: A systematic review of epidemiology, comorbidities and mortality.. J. Prev. Alzheimers Dis..

[4] Panza F., Lozupone M., Logroscino G., Imbimbo B.P. (2019). A critical appraisal of amyloid-β-targeting therapies for Alzheimer disease.. Nat. Rev. Neurol..

[5] Ashraf G.M., Alexiou A. (2019). Biological, Diagnostic and Therapeutic Advances in Alzheimer’s Disease..

[6] Cummings J.L., Gonzalez M.I., Pritchard M.C., May P.C., Toledo-Sherman L.M., Harris G.A. (2023). The therapeutic landscape of tauopathies: Challenges and prospects.. Alzheimers Res. Ther..

[7] Johansson I.L., Samuelsson C., Müller N. (2020). Patients’ and communication partners’ experiences of communicative changes in Parkinson’s disease.. Disabil. Rehabil..

[8] Kim J.S., Hong S.B., Park K.W., Lee A.T.C. (2024). Psychotic symptoms in patients with major neurological diseases.. J. Clin. Neurol..

[9] Zhang X.X., Tian Y., Wang Z.T., Ma Y.H., Tan L., Yu J.T. (2021). The epidemiology of Alzheimer’s disease modifiable risk factors and prevention.. J. Prev. Alzheimers Dis..

[10] Di Battista A.M., Heinsinger N.M., Rebeck G.W. (2016). Alzheimer’s disease genetic risk factor APOE-ε4 also affects normal brain function.. Curr. Alzheimer Res..

[11] Sáiz-Vazquez O., Puente-Martínez A., Pacheco-Bonrostro J., Ubillos-Landa S. (2023). Blood pressure and Alzheimer’s disease: A review of meta-analysis.. Front. Neurol..

[12] Porsteinsson A.P., Isaacson R.S., Knox S., Sabbagh M.N., Rubino I. (2021). Diagnosis of early Alzheimer’s disease: Clinical practice in 2021.. J. Prev. Alzheimers Dis..

[13] Chehrehnegar N., Nejati V., Shati M., Rashedi V., Lotfi M., Adelirad F., Foroughan M. (2020). Early detection of cognitive disturbances in mild cognitive impairment: A systematic review of observational studies.. Psychogeriatrics.

[14] Domínguez-Fernández C., Egiguren-Ortiz J., Razquin J., Gómez-Galán M., De las Heras-García L., Paredes-Rodríguez E., Astigarraga E., Miguélez C., Barreda-Gómez G. (2023). Review of technological challenges in personalised medicine and early diagnosis of neurodegenerative disorders.. Int. J. Mol. Sci..

[15] Kim S.J., Lee H.Y. (2022). *In vivo* molecular imaging in preclinical research.. Lab. Anim. Res..

[16] Mok V.C.T., Pendlebury S., Wong A., Alladi S., Au L., Bath P.M., Biessels G.J., Chen C., Cordonnier C., Dichgans M., Dominguez J., Gorelick P.B., Kim S., Kwok T., Greenberg S.M., Jia J., Kalaria R., Kivipelto M., Naegandran K., Lam L.C.W., Lam B.Y.K., Lee A.T.C., Markus H.S., O’Brien J., Pai M.C., Pantoni L., Sachdev P., Skoog I., Smith E.E., Srikanth V., Suh G.H., Wardlaw J., Ko H., Black S.E., Scheltens P. (2020). Tackling challenges in care of Alzheimer’s disease and other dementias amid the COVID‐19 pandemic, now and in the future.. Alzheimers Dement..

[17] Bogdanovic B., Eftimov T., Simjanoska M. (2022). In-depth insights into Alzheimer’s disease by using explainable machine learning approach.. Sci. Rep..

[18] Kaur S., DasGupta G., Singh S. (2019). Altered neurochemistry in Alzheimer’s disease: Targeting neurotransmitter receptor mechanisms and therapeutic strategy.. Neurophysiology.

[19] Allen Y., Foubert M., Curtin D., Hennessy C., Twomey N., Galvin A., Naughton C. (2022). 323 A multidisciplinary quality improvement project for managing non-cognitive symptoms of dementia in acute care.. Age and Ageing.

[20] Guzzon A., Rebba V., Paccagnella O., Rigon M., Boniolo G. (2023). The value of supportive care: A systematic review of cost-effectiveness of non-pharmacological interventions for dementia.. PLoS One.

[21] Reynolds D.S. (2019). A short perspective on the long road to effective treatments for Alzheimer’s disease.. Br. J. Pharmacol..

[22] Rajasekhar K., Govindaraju T. (2018). Current progress, challenges and future prospects of diagnostic and therapeutic interventions in Alzheimer’s disease.. RSC Advances.

[23] Bertrand D., Wallace T.L. (2020). A review of the cholinergic system and therapeutic approaches to treat brain disorders..

[24] Chen Z.R., Huang J.B., Yang S.L., Hong F.F. (2022). Role of cholinergic signaling in Alzheimer’s disease.. Molecules.

[25] Verma S., Kumar A., Tripathi T., Kumar A. (2018). Muscarinic and nicotinic acetylcholine receptor agonists: Current scenario in Alzheimer’s disease therapy.. J. Pharm. Pharmacol..

[26] Thompson K.J., Tobin A.B. (2020). Crosstalk between the M1 muscarinic acetylcholine receptor and the endocannabinoid system: A relevance for Alzheimer’s disease?. Cell. Signal..

[27] Song X., Jensen M.Ø., Jogini V., Stein R.A., Lee C.H., Mchaourab H.S., Shaw D.E., Gouaux E. (2018). Mechanism of NMDA receptor channel block by MK-801 and memantine.. Nature.

[28] Conway M.E. (2020). Alzheimer’s disease: Targeting the glutamatergic system.. Biogerontology.

[29] Loera-Valencia R., Cedazo-Minguez A., Kenigsberg P.A., Page G., Duarte A.I., Giusti P., Zusso M., Robert P., Frisoni G.B., Cattaneo A., Zille M., Boltze J., Cartier N., Buee L., Johansson G., Winblad B. (2019). Current and emerging avenues for Alzheimer’s disease drug targets.. J. Intern. Med..

[30] Calvo-Flores Guzmán B., Vinnakota C., Govindpani K., Waldvogel H.J., Faull R.L.M., Kwakowsky A. (2018). The GABAergic system as a therapeutic target for Alzheimer’s disease.. J. Neurochem..

[31] Scofield M.D. (2018). Exploring the role of astroglial glutamate release and association with synapses in neuronal function and behavior.. Biol. Psychiatry.

[32] Czapski G.A., Strosznajder J.B. (2021). Glutamate and GABA in microglia-neuron cross-talk in Alzheimer’s disease.. Int. J. Mol. Sci..

[33] Cheng Y.J., Lin C.H., Lane H.Y. (2021). Involvement of cholinergic, adrenergic, and glutamatergic network modulation with cognitive dysfunction in Alzheimer’s disease.. Int. J. Mol. Sci..

[34] Abdi Dezfouli R., Akbariforoud S., Esmaeilidezfouli E. (2024). Are there links between Alzheimer’s disease and ADHD? The efficacy of acetylcholinesterase inhibitors and NMDA receptor antagonists in controlling ADHD symptoms: A systematic review.. Middle East Curr. Psychiatry.

[35] Madav Y., Wairkar S., Prabhakar B. (2019). Recent therapeutic strategies targeting beta amyloid and tauopathies in Alzheimer’s disease.. Brain Res. Bull..

[36] Walsh D.M., Selkoe D.J. (2020). Amyloid β-protein and beyond: The path forward in Alzheimer’s disease.. Curr. Opin. Neurobiol..

[37] Agrawal N., Skelton A.A. (2019). Structure and function of Alzheimer’s amyloid beta proteins from monomer to fibrils: A mini review.. Protein J..

[38] Menendez-Gonzalez M., Padilla-Zambrano H.S., Alvarez G., Capetillo-Zarate E., Tomas-Zapico C., Costa A. (2018). Targeting beta-amyloid at the CSF: A new therapeutic strategy in Alzheimer’s disease.. Front. Aging Neurosci..

[39] Song C., Shi J., Zhang P., Zhang Y., Xu J., Zhao L., Zhang R., Wang H., Chen H. (2022). Immunotherapy for Alzheimer’s disease: Targeting β-amyloid and beyond.. Transl. Neurodegener..

[40] Zhang Y., Chen H., Li R., Sterling K., Song W. (2023). Amyloid β-based therapy for Alzheimer’s disease: Challenges, successes and future.. Signal Transduct. Target. Ther..

[41] Hampel H., Vassar R., De Strooper B., Hardy J., Willem M., Singh N., Zhou J., Yan R., Vanmechelen E., De Vos A., Nisticò R., Corbo M., Imbimbo B.P., Streffer J., Voytyuk I., Timmers M., Tahami Monfared A.A., Irizarry M., Albala B., Koyama A., Watanabe N., Kimura T., Yarenis L., Lista S., Kramer L., Vergallo A. (2021). The β-secretase BACE1 in Alzheimer’s disease.. Biol. Psychiatry.

[42] Moussa-Pacha N.M., Abdin S.M., Omar H.A., Alniss H., Al-Tel T.H. (2020). BACE1 inhibitors: Current status and future directions in treating Alzheimer’s disease.. Med. Res. Rev..

[43] Amirrad F., Bousoik E., Shamloo K., Al-Shiyab H., Nguyen V-H., Montazeri Aliabadi H. (2017). Alzheimer’s disease: Dawn of a new era?. J. Pharm. Pharm. Sci..

[44] Plotkin S.S., Cashman N.R. (2020). Passive immunotherapies targeting Aβ and tau in Alzheimer’s disease.. Neurobiol. Dis..

[45] Gandini A., Bartolini M., Tedesco D., Martinez-Gonzalez L., Roca C., Campillo N.E., Zaldivar-Diez J., Perez C., Zuccheri G., Miti A., Feoli A., Castellano S., Petralla S., Monti B., Rossi M., Moda F., Legname G., Martinez A., Bolognesi M.L. (2018). Tau-centric multitarget approach for Alzheimer’s disease: Development of first-in-class dual glycogen synthase kinase 3β and tau-aggregation inhibitors.. J. Med. Chem..

[46] Su J., Xiao Y., Wei L., Lei H., Sun F., Wang W., Li S., Wang X., Zheng J., Wang J. (2024). A new tau dephosphorylation-targeting chimera for the treatment of tauopathies.. Acta Pharmacol. Sin..

[47] Roth J.R., Rush T., Thompson S.J., Aldaher A.R., Dunn T.B., Mesina J.S., Cochran J.N., Boyle N.R., Dean H.B., Yang Z., Pathak V., Ruiz P., Wu M., Day J.J., Bostwick J.R., Suto M.J., Augelli-Szafran C.E., Roberson E.D. (2024). Development of small-molecule Tau-SH3 interaction inhibitors that prevent amyloid-β toxicity and network hyperexcitability.. Neurotherapeutics.

[48] Gong B., Zhang W., Cong W., Gu Y., Ji W., Yin T., Zhou H., Hu H., Zhuang J., Luo Y., Liu Y., Gao J., Yin Y. (2024). Systemic administration of neurotransmitter‐derived lipidoids‐PROTACs‐DNA nanocomplex promotes Tau clearance and cognitive recovery for Alzheimer’s disease therapy.. Adv. Healthc. Mater..

[49] Muralidar S., Ambi S.V., Sekaran S., Thirumalai D., Palaniappan B. (2020). Role of tau protein in Alzheimer’s disease: The prime pathological player.. Int. J. Biol. Macromol..

[50] Song Y., Dai C.L., Shinohara M., Chyn Tung Y., Zhou S., Huang W.C., Seffouh A., Luo Y., Willadsen M., Jiao Y., Morishima M., Saito Y., Koh S.H., Ortega J., Gong C.X., Lovell J.F. (2024). A pentavalent peptide vaccine elicits Aβ and tau antibodies with prophylactic activity in an Alzheimer’s disease mouse model.. Brain Behav. Immun..

[51] Kaur P., Khera A., Alajangi H.K., Sharma A., Jaiswal P.K., Singh G., Barnwal R.P. (2023). Role of tau in various tauopathies, treatment approaches, and emerging role of nanotechnology in neurodegenerative disorders.. Mol. Neurobiol..

[52] Subhramanyam C.S., Wang C., Hu Q., Dheen S.T. (2019). Seminars in cell & developmental biology..

[53] Leng F., Edison P. (2021). Neuroinflammation and microglial activation in Alzheimer disease: Where do we go from here?. Nat. Rev. Neurol..

[54] Dhapola R., Hota S.S., Sarma P., Bhattacharyya A., Medhi B., Reddy D.H. (2021). Recent advances in molecular pathways and therapeutic implications targeting neuroinflammation for Alzheimer’s disease.. Inflammopharmacology.

[55] Wójtowicz S., Strosznajder A.K., Jeżyna M., Strosznajder J.B. (2020). The novel role of PPAR alpha in the brain: promising target in therapy of Alzheimer’s disease and other neurodegenerative disorders.. Neurochem. Res..

[56] Lepeta K., Lourenco M.V., Schweitzer B.C., Martino A.P.V., Banerjee P., Catuara-Solarz S., de La Fuente Revenga M., Guillem A.M., Haidar M., Ijomone O.M., Nadorp B., Qi L., Perera N.D., Refsgaard L.K., Reid K.M., Sabbar M., Sahoo A., Schaefer N., Sheean R.K., Suska A., Verma R., Vicidomini C., Wright D., Zhang X.D., Seidenbecher C. (2016). Synaptopathies: synaptic dysfunction in neurological disorders – A review from students to students.. J. Neurochem..

[57] Chen Y., Fu A.K.Y., Ip N.Y. (2019). Synaptic dysfunction in Alzheimer’s disease: Mechanisms and therapeutic strategies.. Pharmacol. Ther..

[58] Wang P., Wang F., Ni L., Wu P., Chen J. (2021). Targeting redox-altered plasticity to reactivate synaptic function: A novel therapeutic strategy for cognitive disorder.. Acta Pharm. Sin. B.

[59] DeMattos R.B., Lu J., Tang Y., Racke M.M., DeLong C.A., Tzaferis J.A., Hole J.T., Forster B.M., McDonnell P.C., Liu F., Kinley R.D., Jordan W.H., Hutton M.L. (2012). A plaque-specific antibody clears existing β-amyloid plaques in Alzheimer’s disease mice.. Neuron.

[60] Panza F., Lozupone M., Solfrizzi V., Sardone R., Piccininni C., Dibello V., Stallone R., Giannelli G., Bellomo A., Greco A., Daniele A., Seripa D., Logroscino G., Imbimbo B.P. (2018). BACE inhibitors in clinical development for the treatment of Alzheimer’s disease.. Expert Rev. Neurother..

[61] Fettelschoss A., Zabel F., Bachmann M.F. (2014). Vaccination against Alzheimer disease.. Hum. Vaccin. Immunother..

[62] Crespi G.A.N., Hermans S.J., Parker M.W., Miles L.A. (2015). Molecular basis for mid-region amyloid-β capture by leading Alzheimer’s disease immunotherapies.. Sci. Rep..

[63] Feinberg H., Saldanha J.W., Diep L., Goel A., Widom A., Veldman G.M., Weis W.I., Schenk D., Basi G.S. (2014). Crystal structure reveals conservation of amyloid-β conformation recognized by 3D6 following humanization to bapineuzumab.. Alzheimers Res. Ther..

[64] Ultsch M., Li B., Maurer T., Mathieu M., Adolfsson O., Muhs A., Pfeifer A., Pihlgren M., Bainbridge T.W., Reichelt M., Ernst J.A., Eigenbrot C., Fuh G., Atwal J.K., Watts R.J., Wang W. (2016). Structure of crenezumab complex with Aβ shows loss of β-hairpin.. Sci. Rep..

[65] Silva A.R., Grosso C., Delerue-Matos C., Rocha J.M. (2019). Comprehensive review on the interaction between natural compounds and brain receptors: Benefits and toxicity.. Eur. J. Med. Chem..

[66] Basheer N., Smolek T., Hassan I., Liu F., Iqbal K., Zilka N., Novak P. (2023). Does modulation of tau hyperphosphorylation represent a reasonable therapeutic strategy for Alzheimer’s disease? From preclinical studies to the clinical trials.. Mol. Psychiatry.

[67] Feng Y.S., Tan Z.X., Wu L.Y., Dong F., Zhang F. (2020). The involvement of NLRP3 inflammasome in the treatment of Alzheimer’s disease.. Ageing Res. Rev..

[68] Guo S., Wang H., Yin Y. (2022). Microglia polarization from M1 to M2 in neurodegenerative diseases.. Front. Aging Neurosci..

[69] Ahmad M.H., Fatima M., Mondal A.C. (2019). Influence of microglia and astrocyte activation in the neuroinflammatory pathogenesis of Alzheimer’s disease: Rational insights for the therapeutic approaches.. J. Clin. Neurosci..

[70] Sanz P., Garcia-Gimeno M.A. (2020). Reactive glia inflammatory signaling pathways and epilepsy.. Int. J. Mol. Sci..

[71] Appelbaum L.G., Shenasa M.A., Stolz L., Daskalakis Z. (2023). Synaptic plasticity and mental health: methods, challenges and opportunities.. Neuropsychopharmacology.

[72] Celli R., Santolini I., Van Luijtelaar G., Ngomba R.T., Bruno V., Nicoletti F. (2019). Targeting metabotropic glutamate receptors in the treatment of epilepsy: Rationale and current status.. Expert Opin. Ther. Targets.

[73] Cao J., Hou J., Ping J., Cai D. (2018). Advances in developing novel therapeutic strategies for Alzheimer’s disease.. Mol. Neurodegener..

[74] Liu J., Yang B., Ke J., Li W., Suen W.C. (2016). Antibody-based drugs and approaches against amyloid-β species for Alzheimer’s disease immunotherapy.. Drugs Aging.

[75] Ramanan V.K., Day G.S. (2023). Anti-amyloid therapies for Alzheimer disease: Finally, good news for patients.. Mol. Neurodegener..

[76] Roytman M., Mashriqi F., Al-Tawil K., Schulz P.E., Zaharchuk G., Benzinger T.L.S., Franceschi A.M. (2023). Amyloid-related imaging abnormalities: An update.. AJR Am. J. Roentgenol..

[77] Polanco J.C., Li C., Bodea L.G., Martinez-Marmol R., Meunier F.A., Götz J. (2018). Amyloid-β and tau complexity — towards improved biomarkers and targeted therapies.. Nat. Rev. Neurol..

[78] Chen Y., Yu Y. (2023). Tau and neuroinflammation in Alzheimer’s disease: interplay mechanisms and clinical translation.. J. Neuroinflammation.

[79] Esquer A., Blanc F., Collongues N. (2023). Immunotherapies targeting amyloid and tau protein in alzheimer’s disease: should we move away from diseases and focus on biological targets? A systematic review and expert opinion.. Neurol. Ther..

[80] Ng P.Y., Chang I.S., Koh R.Y., Chye S.M. (2020). Recent advances in tau-directed immunotherapy against Alzheimer’s disease: An overview of pre-clinical and clinical development.. Metab. Brain Dis..

[81] Xia Y., Prokop S., Giasson B.I. (2021). “Don’t Phos Over Tau”: Recent developments in clinical biomarkers and therapies targeting tau phosphorylation in Alzheimer’s disease and other tauopathies.. Mol. Neurodegener..

[82] Guo Y., Li S., Zeng L-H., Tan J. (2022). Tau-targeting therapy in Alzheimer’s disease: Critical advances and future opportunities.. Ageing Neurodegener. Dis..

[83] Sánchez-Sarasúa S., Fernández-Pérez I., Espinosa-Fernández V., Sánchez-Pérez A.M., Ledesma J.C. (2020). Can we treat neuroinflammation in Alzheimer’s disease?. Int. J. Mol. Sci..

[84] Chandra A., Valkimadi P.E., Pagano G., Cousins O., Dervenoulas G., Politis M. (2019). Applications of amyloid, tau, and neuroinflammation PET imaging to Alzheimer’s disease and mild cognitive impairment.. Hum. Brain Mapp..

[85] El Idrissi F., Gressier B., Devos D., Belarbi K. (2021). A computational exploration of the molecular network associated to neuroinflammation in Alzheimer’s Disease.. Front. Pharmacol..

[86] Foster J.B., Lashley R., Zhao F., Wang X., Kung N., Askwith C.C., Lin L., Shultis M.W., Hodgetts K.J., Lin C.L.G. (2019). Enhancement of tripartite synapses as a potential therapeutic strategy for Alzheimer’s disease: A preclinical study in rTg4510 mice.. Alzheimers Res. Ther..

[87] Zhou H., Li H., Gowravaram N., Quan M., Kausar N., Gomperts S.N. (2022). Disruption of hippocampal neuronal circuit function depends upon behavioral state in the APP/PS1 mouse model of Alzheimer’s disease.. Sci. Rep..

[88] Cong Y.F., Liu F.W., Xu L., Song S.S., Shen X.R., Liu D., Hou X.Q., Zhang H.T. (2023). Rolipram ameliorates memory deficits and depression-like behavior in APP/PS1/tau triple transgenic mice: Involvement of neuroinflammation and apoptosis* via* cAMP signaling.. Int. J. Neuropsychopharmacol..

[89] Meftah S., Gan J. (2023). Alzheimer’s disease as a synaptopathy: Evidence for dysfunction of synapses during disease progression.. Front. Synaptic Neurosci..

[90] Yadollahikhales G., Rojas J.C. (2023). Anti-amyloid immunotherapies for Alzheimer’s disease: A 2023 clinical update.. Neurotherapeutics.

[91] van Bokhoven P., de Wilde A., Vermunt L., Leferink P.S., Heetveld S., Cummings J., Scheltens P., Vijverberg E.G.B. (2021). The Alzheimer’s disease drug development landscape.. Alzheimers Res. Ther..

[92] Kiraly M., Foss J.F., Giordano T. (2023). Neuroinflammation, its role in Alzheimer’s disease and therapeutic strategies.. J. Prev. Alzheimers Dis..

[93] Zhao K., Li Z., Liu Q., Cheng Y., Barreto G.E., Liu R. (2022). Editorial: Novel therapeutic target and drug discovery for neurological diseases.. Front. Pharmacol..

[94] Spagnuolo C., Moccia S., Russo G.L. (2018). Anti-inflammatory effects of flavonoids in neurodegenerative disorders.. Eur. J. Med. Chem..

[95] Nisticò R., Pignatelli M., Piccinin S., Mercuri N.B., Collingridge G. (2012). Targeting synaptic dysfunction in Alzheimer’s disease therapy.. Mol. Neurobiol..

[96] Huang L.K., Kuan Y.C., Lin H.W., Hu C.J. (2023). Clinical trials of new drugs for Alzheimer disease: A 2020-2023 update.. J. Biomed. Sci..

[97] Hampel H., Williams C., Etcheto A., Goodsaid F., Parmentier F., Sallantin J., Kaufmann W.E., Missling C.U., Afshar M. (2020). A precision medicine framework using artificial intelligence for the identification and confirmation of genomic biomarkers of response to an Alzheimer’s disease therapy: Analysis of the blarcamesine (ANAVEX2‐73) Phase 2a clinical study.. Alzheimers Dement. (N. Y.).

[98] Rashad A., Rasool A., Shaheryar M., Sarfraz A., Sarfraz Z., Robles-Velasco K., Cherrez-Ojeda I. (2022). Donanemab for Alzheimer’s disease: A systematic review of clinical trials. Healthcare..

[99] Krafft G.A., Jerecic J., Siemers E., Cline E.N. (2022). ACU193: an immunotherapeutic poised to test the amyloid β oligomer hypothesis of Alzheimer’s disease.. Front. Neurosci..

[100] Shulman M., Kong J., O’Gorman J., Ratti E., Rajagovindan R., Viollet L., Huang E., Sharma S., Racine AM., Czerkowicz J. (2023). TANGO: A placebo-controlled randomized phase 2 study of efficacy and safety of the anti-tau monoclonal antibody gosuranemab in early Alzheimer’s disease.. Nat. Aging.

[101] Coimbra J.R.M., Resende R., Custódio J.B.A., Salvador J.A.R., Santos A.E. (2024). BACE1 inhibitors for Alzheimer’s disease: Current challenges and future perspectives.. J. Alzheimers Dis..

[102] Sivanantharajah L., Mudher A. (2022). Curcumin as a holistic treatment for tau pathology.. Front. Pharmacol..

[103] Thal L.J., Ferris S.H., Kirby L., Block G.A., Lines C.R., Yuen E., Assaid C., Nessly M.L., Norman B.A., Baranak C.C., Reines S.A. (2005). A randomized, double-blind, study of rofecoxib in patients with mild cognitive impairment.. Neuropsychopharmacology.

[104] Menon S.N., Zerin F., Ezewudo E., Simon N.P., Menon S.N., Daniel M.L., Green A.J., Pandey A., Mackay C.E., Hafez S., Moniri N.H., Hasan R. (2023). Neflamapimod inhibits endothelial cell activation, adhesion molecule expression, leukocyte attachment and vascular inflammation by inhibiting p38 MAPKα and NF-κB signaling.. Biochem. Pharmacol..

[105] Shen B., Lin Y., Bi C., Zhou S., Bai Z., Zheng G., Zhou J. (2019). Translational informatics for Parkinson’s disease: From big biomedical data to small actionable alterations.. Genomics Proteomics Bioinformatics.

[106] Zahra M.A., Al-Taher A., Alquhaidan M., Hussain T., Ismail I., Raya I., Kandeel M. (2024). The synergy of artificial intelligence and personalized medicine for the enhanced diagnosis, treatment, and prevention of disease.. Drug Metab. Pers. Ther..

[107] Gold M., Amatniek J., Carrillo M.C., Cedarbaum J.M., Hendrix J.A., Miller B.B., Robillard J.M., Rice J.J., Soares H., Tome M.B., Tarnanas I., Vargas G., Bain L.J., Czaja S.J. (2018). Digital technologies as biomarkers, clinical outcomes assessment, and recruitment tools in Alzheimer’s disease clinical trials.. Alzheimers Dement. (N. Y.).

[108] Ahangari N., Fischer C.E., Schweizer T.A., Munoz D.G. (2023). Cognitive resilience and severe Alzheimer’s disease neuropathology.. Aging Brain.

[109] Al-Ayyad M., Owida H.A., De Fazio R., Al-Naami B., Visconti P. (2023). Electromyography monitoring systems in rehabilitation: A review of clinical applications, wearable devices and signal acquisition methodologies.. Electronics (Basel).

[110] Lott S.A., Streel E., Bachman S.L., Bode K., Dyer J., Fitzer-Attas C., Goldsack J.C., Hake A., Jannati A., Fuertes R.S., Fromy P. (2024). Digital health technologies for Alzheimer’s disease and related dementias: Initial results from a landscape analysis and community collaborative effort.. J. Prev. Alzheimers Dis..

[111] Owens A.P., Ballard C., Beigi M., Kalafatis C., Brooker H., Lavelle G., Brønnick K.K., Sauer J., Boddington S., Velayudhan L., Aarsland D. (2020). Implementing remote memory clinics to enhance clinical care during and after COVID-19.. Front. Psychiatry.

[112] Gomez-Valades A., Martinez-Tomas R., Rincon M. (2021). Integrative base ontology for the research analysis of Alzheimer’s disease-related mild cognitive impairment.. Front. Neuroinform..

[113] Poldrack R.A., Yarkoni T. (2016). From brain maps to cognitive ontologies: informatics and the search for mental structure.. Annu. Rev. Psychol..

[114] Alobaidi M., Malik K.M., Hussain M. (2018). Automated ontology generation framework powered by linked biomedical ontologies for disease-drug domain.. Comput. Methods Programs Biomed..

[115] Swan K., Speyer R., Scharitzer M., Farneti D., Brown T., Woisard V., Cordier R. (2023). Measuring what matters in healthcare: A practical guide to psychometric principles and instrument development.. Front. Psychol..

[116] Malhotra A., Younesi E., Gündel M., Müller B., Heneka M.T., Hofmann-Apitius M. (2014). ADO: A disease ontology representing the domain knowledge specific to Alzheimer’s disease.. Alzheimers Dement..

[117] Timón-Reina S., Rincón M., Martínez-Tomás R., Kirsebom B.E., Fladby T. (2023). A knowledge graph framework for dementia research data.. Appl. Sci. (Basel).

[118] Shoaip N., Barakat S., Elmogy M. Alzheimer’s Disease Integrated Ontology (ADIO).. 14th International Conference on Computer Engineering and Systems (ICCES).

[119] Berros N., El Mendili F., Filaly Y., El Bouzekri El Idrissi Y. (2023). Enhancing digital health services with big data analytics.. Big Data Cogn. Comput..

[120] Mahendran N. (2022). P M, D.R.V. A deep learning framework with an embedded-based feature selection approach for the early detection of the Alzheimer’s disease.. Comput. Biol. Med..

[121] Uspenskaya-Cadoz O., Alamuri C., Wang L., Yang M., Khinda S., Nigmatullina Y., Cao T., Kayal N., O’Keefe M., Rubel C. (2019). Machine learning algorithm helps identify non-diagnosed prodromal Alzheimer’s disease patients in the general population.. J. Prev. Alzheimers Dis..

[122] Singhania U., Tripathy B., Hasan M.K., Anumbe N.C., Alboaneen D., Ahmed F.R.A., Ahmed T.E., Nour M.M.M. (2021). A predictive and preventive model for onset of Alzheimer’s disease.. Front. Public Health.

[123] Uddin K.M.M., Alam M.J., Uddin M.A., Aryal S. (2023). a novel approach utilizing machine learning for the early diagnosis of Alzheimer’s disease.. Biomed. Materials & Devices.

[124] Kavitha C., Mani V., Srividhya S.R., Khalaf O.I., Tavera Romero C.A. (2022). Early-stage Alzheimer’s disease prediction using machine learning models.. Front. Public Health.

[125] Abrol A., Fu Z., Du Y., Calhoun V.D. (2019). Multimodal data fusion of deep learning and dynamic functional connectivity features to predict Alzheimer’s disease progression.. In 2019 41st annual international conference of the IEEE engineering in medicine and biology society (EMBC)..

[126] Bi X., Cai R., Wang Y., Liu Y. (2019). Effective diagnosis of Alzheimer’s disease* via* multimodal fusion analysis framework.. Front. Genet..

[127] Rahim N., El-Sappagh S., Ali S., Muhammad K., Del Ser J., Abuhmed T. (2023). Prediction of Alzheimer’s progression based on multimodal Deep-Learning-based fusion and visual explainability of time-series data.. Inf. Fusion.

[128] Bellantuono L., Monaco A., Amoroso N., Lacalamita A., Pantaleo E., Tangaro S., Bellotti R. (2022). Worldwide impact of lifestyle predictors of dementia prevalence: An explainable Artificial Intelligence analysis.. Frontiers in. Big Data.

[129] El-Sappagh S., Alonso J.M., Islam S.M.R., Sultan A.M., Kwak K.S. (2021). A multilayer multimodal detection and prediction model based on explainable artificial intelligence for Alzheimer’s disease.. Sci. Rep..

[130] Jahan S., Abu Taher K., Kaiser M.S., Mahmud M., Rahman M.S., Hosen A.S.M.S., Ra I.H. (2023). Explainable AI-based Alzheimer’s prediction and management using multimodal data.. PLoS One.

[131] Saleh H., ElRashidy N., Abd Elaziz M. (2024). Genetic algorithm-based hybrid deep learning model for explainable Alzheimer’s disease prediction using temporal multimodal cognitive data.. Int. J. Data Sci. Anal..

[132] Cummings J., Aisen P.S., DuBois B., Frölich L., Jack C.R., Jones R.W., Morris J.C., Raskin J., Dowsett S.A., Scheltens P. (2016). Drug development in Alzheimer’s disease: The path to 2025.. Alzheimers Res. Ther..

[133] Cavalli A., Bolognesi M.L., Minarini A., Rosini M., Tumiatti V., Recanatini M., Melchiorre C. (2008). Multi-target-directed ligands to combat neurodegenerative diseases.. J. Med. Chem..

[134] Kabir M.T., Uddin M.S., Mamun A.A., Jeandet P., Aleya L., Mansouri R.A., Ashraf G.M., Mathew B., Bin-Jumah M.N., Abdel-Daim M.M. (2020). Combination drug therapy for the management of Alzheimer’s disease.. Int. J. Mol. Sci..

[135] Gharat R., Dixit G., Khambete M., Prabhu A. (2024). Targets, trials and tribulations in Alzheimer therapeutics.. Eur. J. Pharmacol..

[136] Mukherjee A., Biswas S., Roy I. (2024). Immunotherapy: An emerging treatment option for neurodegenerative diseases.. Drug Discov. Today.

[137] Emens L.A., Ascierto P.A., Darcy P.K., Demaria S., Eggermont A.M.M., Redmond W.L., Seliger B., Marincola F.M. (2017). Cancer immunotherapy: Opportunities and challenges in the rapidly evolving clinical landscape.. Eur. J. Cancer.

[138] Alam J., Sharma L. (2019). Potential enzymatic targets in Alzheimer’s: A comprehensive review.. Curr. Drug Targets.

[139] Okano H., Miyawaki A., Kasai K. (2015). Brain/MINDS: Brain-mapping project in Japan.. Philos. Trans. R. Soc. Lond. B Biol. Sci..

[140] Roman V., Viktor S. (2022). The role of big brain science in the development of artificial intelligence technologies.. Arch. Venez. Farmacol. Ter..

[141] Gosztyla M.L., Brothers H.M., Robinson S.R. (2018). Alzheimer’s amyloid-β is an antimicrobial peptide: A review of the evidence.. J. Alzheimers Dis..

[142] Anderson G. (2023). Why do anti-amyloid beta antibodies not work? Time to reconceptualize dementia pathophysiology by incorporating astrocyte melatonergic pathway desynchronization from amyloid-beta production Braz.. J. Psychiatry.

[143] Markus R.P., Fernandes P.A., Kinker G.S., da Silveira Cruz-Machado S., Marçola M. (2018). Immune‐pineal axis – acute inflammatory responses coordinate melatonin synthesis by pinealocytes and phagocytes.. Br. J. Pharmacol..

[144] Muxel S.M., Pires-Lapa M.A., Monteiro A.W.A., Cecon E., Tamura E.K., Floeter-Winter L.M., Markus R.P. (2012). NF-κB drives the synthesis of melatonin in RAW 264.7 macrophages by inducing the transcription of the arylalkylamine-N-acetyltransferase (AA-NAT) gene.. PLoS One.

[145] Calandria J.M., Do K.V., Kala-Bhattacharjee S., Obenaus A., Belayev L., Bazan N.G. (2023). cRel and Wnt5a/Frizzled 5 receptor-mediated inflammatory regulation reveal novel neuroprotectin D1 targets for neuroprotection.. Cell. Mol. Neurobiol..

[146] Chen D., Lan G., Li R., Mei Y., Shui X., Gu X., Wang L., Zhang T., Gan C.L., Xia Y., Hu L., Tian Y., Zhang M., Lee T.H. (2022). Melatonin ameliorates tau-related pathology* via* the miR-504-3p and CDK5 axis in Alzheimer’s disease.. Transl. Neurodegener..

[147] Terao I., Kodama W. (2024). Comparative efficacy, tolerability, and acceptability of donanemab, lecanemab, aducanumab, melatonin, and aerobic exercise for a short time on cognitive function in mild cognitive impairment and mild Alzheimer’s disease: A systematic review and network meta-analysis.. J. Alzheimers Dis..

